# Tbx1 controls the morphogenesis of pharyngeal pouch epithelia through mesodermal Wnt11r and Fgf8a

**DOI:** 10.1242/dev.111740

**Published:** 2014-09

**Authors:** Chong Pyo Choe, J. Gage Crump

**Affiliations:** Broad California Institute of Regenerative Medicine Center, Department of Stem Cell Biology and Regenerative Medicine, Keck School of Medicine, University of Southern California, Los Angeles, CA 90033, USA

**Keywords:** Epithelial morphogenesis, Fgf8, Pharyngeal pouches, Tbx1, Wnt11r, Zebrafish

## Abstract

The pharyngeal pouches are a segmental series of epithelial structures that organize the embryonic vertebrate face. In mice and zebrafish that carry mutations in homologs of the DiGeorge syndrome gene *TBX1*, a lack of pouches correlates with severe craniofacial defects, yet how Tbx1 controls pouch development remains unclear. Using mutant and transgenic rescue experiments in zebrafish, we show that Tbx1 functions in the mesoderm to promote the morphogenesis of pouch-forming endoderm through *wnt11r* and *fgf8a* expression. Consistently, compound losses of *wnt11r* and *fgf8a* phenocopy *tbx1* mutant pouch defects, and mesoderm-specific restoration of Wnt11r and Fgf8a rescues *tbx1* mutant pouches. Time-lapse imaging further reveals that Fgf8a acts as a Wnt11r-dependent guidance cue for migrating pouch cells. We therefore propose a two-step model in which Tbx1 coordinates the Wnt-dependent epithelial destabilization of pouch-forming cells with their collective migration towards Fgf8a-expressing mesodermal guideposts.

## INTRODUCTION

From the embryonic endoderm emerge a number of epithelial outpocketings that generate the initial anlagen of diverse organs. In the head, these include a series of pharyngeal pouches that guide the development of neural-crest-derived cells into bone and cartilage and fuse with ectodermal clefts to generate the gills, thymus, parathyroid and other structures (Crump et al., 2004a; Piotrowski et al., 2003; Schwend and Ahlgren, 2009). Although clearly crucial for vertebrate head development, how pouches achieve their stereotyped positions and morphologies remains poorly understood.

Given the importance of pouches in craniofacial development, it is not surprising that mutations in genes that regulate their morphogenesis are implicated in human birth defects. DiGeorge syndrome, commonly associated with heterozygous deletion of a region of chromosome 22 containing the *TBX1* gene, is characterized by defects in the facial skeleton, thymus and heart (Lindsay et al., 2001). It is thought that DiGeorge defects can be attributed, at least in part, to earlier malformations of pouches (Epstein, 2001). In support of this, the zebrafish *van gogh* mutant, which is mutated for *tbx1*, has severe defects in pouch formation and facial skeletal development (Piotrowski and Nusslein-Volhard, 2000; Piotrowski et al., 2003). Similarly, *Tbx1* mutant mice lack pouches and have many of the craniofacial and organ defects of DiGeorge individuals (Vitelli et al., 2002a). *Tbx1* is expressed in both the pre-pouch endoderm and surrounding mesoderm; however, the tissue requirements for Tbx1 in pouch development, as well as its crucial downstream targets, remain controversial. In one study, it has been shown that conditional deletion of *Tbx1* in the *Mesp1*-positive mesoderm of mice results in pouch defects, and re-introduction of *Tbx1* into *Mesp1*-positive mesoderm rescues pouches (Zhang et al., 2006). However, another study concludes that deletion of *Tbx1* in the *Foxg1*-positive endoderm likewise results in defective pouch outgrowth (Arnold et al., 2006). In zebrafish, transplantation of wild-type endoderm has been reported to rescue some of the facial cartilage defects of *tbx1* mutants, yet the observed rescue is mild and restoration of pouch morphology was not examined (Piotrowski et al., 2003). By using a transgenic strategy to reintroduce Tbx1 and its putative effectors into the mesoderm and/or endoderm of zebrafish, we show here that Tbx1 has crucial functions in the mesoderm for pouch morphogenesis yet is dispensable for the earlier segmentation of the pouch-forming endoderm.

We have previously identified a role for the *nkx2.5*-expressing subpopulation of mesoderm in guiding pouch formation, with genetic ablation of this mesoderm resulting in pouch defects similar to those of *tbx1* mutants (Choe et al., 2013). One common target of Tbx1 in the mesoderm of mice and fish might be Fgf8. In mice, mesoderm-specific deletion of *Tbx1* results in loss of *Fgf8* expression from the anterior heart field mesoderm (Zhang et al., 2006), and heterozygosity of *Fgf8* enhances DiGeorge-like defects in *Tbx1* heterozygous animals (Vitelli et al., 2002b). In zebrafish, loss of *fgf8a*, in particular in combination with reduction of Fgf3 levels, results in severe pouch defects that can be partially rescued by wild-type mesoderm transplants; similar pouch defects are seen upon global inhibition of Fgf signaling with the drug SU5402 (Crump et al., 2004a). However, insertion of *Fgf8* into the *Tbx1* locus fails to rescue *Tbx1* mutant defects (Vitelli et al., 2006). Thus, loss of Fgf8 alone cannot account for the severe pouch defects seen in *Tbx1* mutants. Indeed, a number of other potential targets of Tbx1, such as *Pitx2* (Nowotschin et al., 2006), *Crk1* (Guris et al., 2001), retinoic acid (Roberts et al., 2006; Zhang et al., 2006), Vegf signaling (Stalmans et al., 2003) and Tgfβ signaling (Wurdak et al., 2005) have been identified, yet how they might interact with Tbx1 to control craniofacial morphogenesis remains poorly understood.

Although canonical Wnt signaling (i.e. nuclear β-catenin) has been proposed to negatively regulate *Tbx1* expression (Huh and Ornitz, 2010), less is known about potential roles of Wnt signaling downstream of Tbx1. In zebrafish, we have previously reported crucial roles for cytoplasmic (i.e. non-canonical) Wnt signaling in orchestrating distinct steps of pouch morphogenesis (Choe et al., 2013). Pouches develop through a transient multi-layering of endodermal epithelial cells, followed by their lateral collective migration and reorganization into mature bilayers. Mesoderm-derived Wnt11r promotes the loss of columnar morphology that accompanies the multi-layering process, whereas ectodermal Wnt4a promotes the reacquisition of columnar morphology during bilayer maturation. Here, we show that Tbx1 is required for the expression of *wnt11r* in the developing mesoderm, with Wnt11r acting together with Fgf8a to coordinate pouch morphogenesis. *In vivo* time-lapse imaging further reveals how Wnt11r-dependent destabilization of the pre-pouch endoderm allows epithelial cells to collectively migrate towards sources of Fgf8a in the adjacent mesoderm. Taken together, the data from this study reveal how two targets of Tbx1 in the mesoderm – Wnt11r and Fgf8a – coordinate the morphogenesis of growing pouches.

## RESULTS

### Tbx1 is required for the morphogenesis but not the initial segmentation of pouch-forming endoderm

**Fig. 1.**
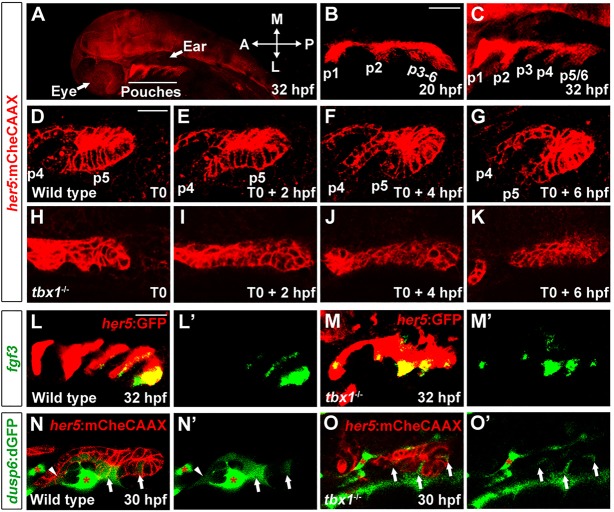
**Requirement of Tbx1 for pouch morphogenesis.** (A) A zoomed out view of a 32 hpf *her5*:mCherryCAAX (red) embryo shows the position of pouches relative to the developing eye and ear. (B,C) The first two pouches (p1, p2) form by 20 hpf, with the remaining pouches (p3-p6) developing at one per 4 h. (D-K) Still images from time-lapse confocal recordings of wild-type and *tbx1* mutant pouch development at the times indicated (bottom right of the images) (see supplementary material Movie 1A,B). *her5*:mCherryCAAX labels endodermal cell membranes. Recordings were started at 26 hpf (T0), a stage when the fourth and fifth pouches (p4 and p5) are beginning to develop in the wild-type example. Compared with the typical pouch outpocketing behavior seen in all three wild-type siblings, no endodermal outpocketing was observed in three out of three *tbx1* mutants (H-K). (L,M) Fluorescent *in situ* hybridization for *fgf3* (green) and GFP immunohistochemistry to label the *her5*:GFP-positive pouch endoderm (red). *fgf3* was expressed in a segmental pattern in all 39 wild-type siblings and all 14 *tbx1* mutants. (N,O) Confocal sections of pre-pouch endoderm in *dusp6*:dGFP; *her5*:mCherryCAAX transgenic embryos. In wild type, *dusp6*:dGFP (green) was expressed segmentally in already formed pouches (arrowhead) and in clusters of presumptive pouch endoderm before outpocketing (arrows); strong expression was also seen in adjacent mesoderm (asterisks) (*n*=18). In *tbx1* mutants, *dusp6*:dGFP was expressed at lower levels and in fewer cells, yet a segmental pattern in the endoderm was still detected (*n*=7). Note that we increased the relative gain of the green channel in *tbx1* mutants to reveal the weak segmental *dusp6*:dGFP expression. See supplementary material Fig. S1 for the unaltered images. L′-O′ show the green channel alone. Scale bars: 40 μm (B,C,L,M); 20 μm (D-K,N,O).

**Fig. 2.**
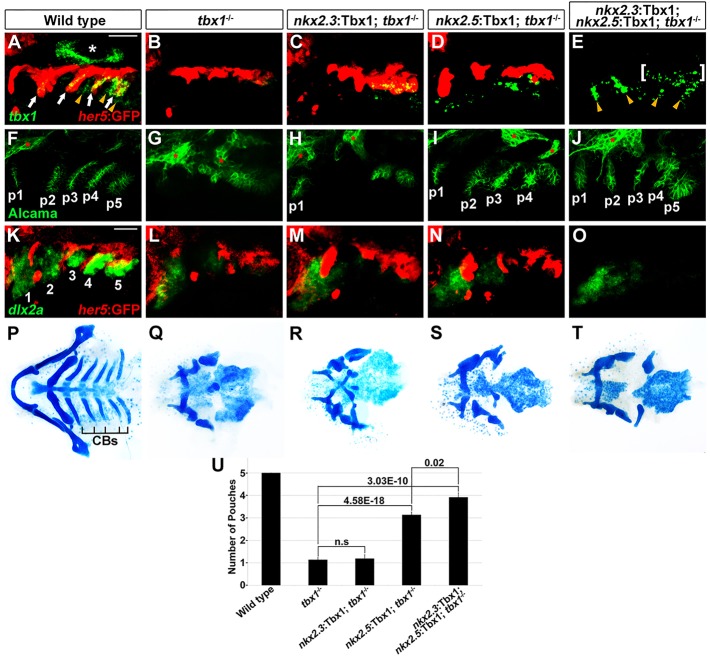
**Tbx1 is required in the *nkx2.5*-positive mesoderm for pouch development.** (A-E) Fluorescent *in situ* hybridization for *tbx1* (green) and GFP immunohistochemistry to detect *her5*:GFP-positive endoderm (red) at 34 hpf. In all 58 wild-type siblings, *tbx1* expression was observed in *her5*:GFP-positive endoderm (arrows), adjacent mesoderm (arrowheads) and the ear (asterisk). In all 21 *tbx1* mutants, all *tbx1* expression was lost. *tbx1* expression was selectively restored in the *her5*:GFP-positive endoderm of all 17 *nkx2.3*:Tbx1; *tbx1*^−/−^ embryos and in the mesoderm of all nine *nkx2.5*:Tbx1; *tbx1*^−/−^ embryos. Inclusion of both transgenes restored *tbx1* expression over a broader area (brackets for presumptive endoderm and arrowheads for presumptive mesoderm) in all four *nkx2.3*:Tbx1; *nkx2.5*:Tbx1; *tbx1*^−/−^ embryos, although for technical reasons *her5*:GFP was not included. (F-J) In wild-type zebrafish at 34 hpf, immunohistochemistry for Alcama (green) labeled five pouches (p1-p5). *nkx2.5*:Tbx1; *tbx1*^−/−^ and *nkx2.3*:Tbx1; *nkx2.5*:Tbx1; *tbx1*^−/−^ embryos displayed partial rescue of pouches compared with *tbx1* mutants, whereas *nkx2.3*:Tbx1; *tbx1*^−/−^ embryos did not. Sensory ganglia are indicated with red asterisks. (K-O) Fluorescent *in situ* hybridization for *dlx2a* (green) and GFP immunohistochemistry for *her5*:GFP (red) at 30 hpf. In wild-type zebrafish, *dlx2a* was expressed in the neural-crest-derived mesenchyme of each arch (numbered), with higher expression in posterior arches. In all *tbx1*^−/−^ (*n*=9), *nkx2.3*:Tbx1; *tbx1*^−/−^ (*n*=6), *nkx2.5*:Tbx1; *tbx1*^−/−^ (*n*=4) and *nkx2.3*:Tbx1; *nkx2.5*:Tbx1; *tbx1*^−/−^ (*n*=4) embryos, *dlx2a* expression was reduced in the second arch and lost from the more posterior arches. For technical reasons, *her5*:GFP was not included in O. (P-T) Ventral whole-mount views of dissected facial cartilages. Wild-type zebrafish (P) invariantly formed five ceratobranchials (CBs) on each side. CBs were missing in all *tbx1* mutants (Q, *n*=41) and not recovered in *nkx2.3*:Tbx1; *tbx1*^−/−^ (R, *n*=23), *nkx2.5*:Tbx1; *tbx1*^−/−^ (S, *n*=26) or *nkx2.3*:Tbx1; *nkx2.5*:Tbx1; *tbx1*^−/−^ (T, *n*=24) embryos. (U) Quantification of pouch defects as assessed by Alcama staining in wild-type zebrafish (*n*=342), *tbx1*^−/−^ (*n*=63), *nkx2.3*:Tbx1; *tbx1*^−/−^ (*n*=54), *nkx2.5*:Tbx1; *tbx1*^−/−^ (*n*=22) and *nkx2.3*:Tbx1; *nkx2.5*:Tbx1; *tbx1*^−/−^ (*n*=23). Data represent mean±s.e.m., *P* values are shown. n.s., not significant. Scale bars: 40 μm (A-O).

Tbx1 is clearly required for pouch formation across vertebrates, but its roles in specifying pouch-forming regions in the pharyngeal endoderm versus promoting their later morphogenesis has remained unclear. In order to address the requirements for Tbx1, we first performed time-lapse imaging of pouch cell behavior in *tbx1* mutant zebrafish harboring a *her5*:mCherryCAAX transgene that labels endodermal cell membranes (Choe et al., 2013). Compared with wild-type siblings that showed outpocketing of a new pouch every 4 h (Fig. 1A-G; supplementary material Movie 1A), *her5*:mCherryCAAX-positive endodermal cells were present in *tbx1* mutants yet failed to display outpocketing behavior within the 10-h timeframe of the recordings (Fig. 1H-K; supplementary material Movie 1B). As previously reported (Piotrowski and Nusslein-Volhard, 2000), *tbx1* mutants also retained expression of the immunoglobulin-domain protein Alcama, a marker of maturing pouches, despite the absence of morphological pouches (Fig. 2G). These data support the notion that Tbx1 is required for the morphogenesis but not generation of pouch-forming endoderm.

We next investigated whether the failure to initiate pouch outpocketing in *tbx1* mutants reflects a loss of segmental character in the pre-pouch endoderm, or instead defects in the morphogenesis of pre-specified segmental units. In wild-type zebrafish, an early marker of developing pouches is *fgf3* expression (Fig. 1L) (David et al., 2002; Nissen et al., 2003). Remarkably, despite the near complete loss of morphological pouches, we still observed segmental expression of *fgf3* in the presumptive pouch-forming endoderm of *tbx1* mutants (Fig. 1M). To further explore dynamic Fgf signaling in *tbx1* mutants, we also examined a *dusp6*:dGFP transgenic line in which a destabilized GFP protein is expressed under control of the Fgf target gene *dusp6* (Molina et al., 2007). As with *fgf3* itself, we found that *dusp6*:dGFP marks nascent pouch-forming segments in wild type, with fluorescence apparent in epithelial cell clusters up to two segmental units preceding pouch formation, as well as in adjacent mesoderm (Fig. 1N). *dusp6*:dGFP intensity was diminished in *tbx1* mutants (supplementary material Fig. S1), but enhancement of the fluorescent signal revealed segmental expression within the presumptive pouch-forming endoderm (Fig. 1O). Taken together, the retention of segmental *fgf3* expression and Fgf reporter activity in *tbx1* mutant endoderm indicates that Tbx1 is dispensable for at least some aspects of pre-pouch segmentation.

### Mesodermal Tbx1 is sufficient for pouch morphogenesis but not cartilage formation

As *tbx1* was expressed in both the pharyngeal endoderm and mesoderm (Fig. 2A; supplementary material Fig. S2A-C) (Piotrowski et al., 2003), we investigated the requirements of Tbx1 in each tissue for pouch morphogenesis. To do so, we used an endodermal *nkx2.3* (Choe et al., 2013) or mesodermal *nkx2.5* (Witzel et al., 2012) promoter to restore Tbx1 function in each tissue or both. *In situ* hybridization in combination with *her5*:GFP labeling of endoderm confirmed that stably integrated *nkx2.3*:Tbx1 and *nkx2.5*:Tbx1 transgenes selectively restored *tbx1* expression to the endoderm and mesoderm, respectively, of *tbx1* mutants – although *nkx2.3*:Tbx1 restored *tbx1* expression to lower levels than those in wild type and in only the posterior pouch-forming endoderm (Fig. 2B-E; supplementary material Fig. S2I). *nkx2.3*:Tbx1 and *nkx2.5*:Tbx1 animals were healthy and viable with no pouch defects on their own (supplementary material Fig. S2D-F). Supporting a role for Tbx1 in the mesoderm, *nkx2.5*:Tbx1 was able to partially restore pouch formation in *tbx1* mutants. Although *nkx2.3*:Tbx1 alone did not restore pouches to a significant extent, both transgenes together increased the average number of pouches over *nkx2.*5:Tbx1 alone, indicating that Tbx1 functions in both the mesoderm and endoderm for pouch formation (Fig. 2J).

Surprisingly, despite the robust rescue of pouches by combined *nkx2.5*:Tbx1 and *nkx2.3*:Tbx1 transgenes, we failed to observe any recovery of the ceratobranchial cartilages that depend on pouches for their development (Fig. 2P-T). This inability to rescue cartilage formation might be due to earlier requirements for Tbx1 in neural crest development. In *tbx1* mutants at 36 h post-fertilization (hpf), we observed severe reductions in pharyngeal arch expression of *dlx2a*, a marker of neural-crest-derived skeletogenic ectomesenchyme, particularly in the branchial arches that generate the ceratobranchial cartilages (Fig. 2L). Branchial arch reduction of *dlx2a* was seen as early as 16.5 hpf, and more so at 18 hpf, stages that are well in advance of formation of the third through sixth pouches (supplementary material Fig. S2L-O). Consistent with the lack of cartilage rescue, *dlx2a* expression was also not restored by *nkx2.5*:Tbx1 and *nkx2.3*:Tbx1 transgenes (Fig. 2M-O). Our findings differ somewhat from those of Piotrowski and Nusslein-Volhard (Piotrowski and Nusslein-Volhard, 2000), which found no change in *dlx2a* expression in 18 hpf *tbx1* mutants (although reduced *dlx2a* was reported at 33 hpf). However, we note that the skeletal defects observed in our *tbx1* mutant line are more severe than those presented in this earlier study. Furthermore, the reduction in *dlx2a* expression did not appear to be due to earlier defects in neural crest specification or migration. Cranial neural crest cells expressing *sox10* were present in normal numbers in *tbx1* mutant embryos at 12 hpf (supplementary material Fig. S2J,K), and *sox10*:GFP-positive neural-crest-derived cells still populated the arches of *tbx1* mutants at 30 hpf (supplementary material Fig. S2Q,S). We did detect a modest increase in cell death (as determined using LysoTracker Red staining), both in the arches and throughout the head of *tbx1* mutants, yet proliferation was unaffected (as determined using BrdU staining; supplementary material Fig. S2Q,S,T). Our data indicate an important function of Tbx1 in the *nkx2.5*-positive mesoderm for pouch morphogenesis; however, it is likely that Tbx1 has other requirements for the identity and/or survival of skeletogenic ectomesenchyme.

### Tbx1 is required for mesodermal expression of *wnt11r* and *fgf8a*

**Fig. 3.**
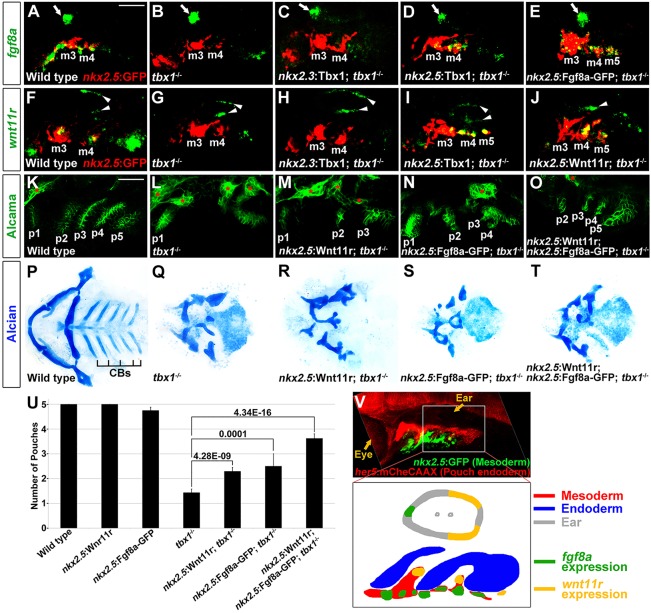
**Rescue of *tbx1^−/−^*pouch defects with mesodermal Fgf8a and Wnt11r.** (A-J) Fluorescent *in situ* hybridization for *fgf8a* or *wnt11r* (green) and GFP immunohistochemistry to detect *nkx2.5*:GFP-positive mesoderm (red) at 30 hpf. In wild type, *fgf8a* was expressed in ventral *nkx2.5*:GFP-positive mesodermal cores of arches 3 and 4 (m3 and m4), whereas *wnt11r* was in more dorsal subsets of these mesodermal cores. In *tbx1* mutants, mesodermal expression of *fgf8a* (*n*=12 of 12) and *wnt11r* (*n*=10 of 11) was lost, although *fgf8a* and *wnt11r* expression in the anterior (arrow) and posterior (arrowheads) portions of the otic vesicle was unaffected. An *nkx2.3*:Tbx1 transgene did not restore mesodermal *fgf8a* (*n*=0 of 7) and *wnt11r* expression (*n*=0 of 5), whereas an *nkx2.5*:Tbx1 transgene restored mesodermal *fgf8a* (*n*=5 of 6) and *wnt11r* expression (*n*=9 of 9). Also, *nkx2.5*:Fgf8-GFP and *nkx2.5*:Wnt11r transgenes restored *fgf8a* (*n*=27 of 27) and *wnt11r* (*n*=21 of 21) expression, respectively. (K-O) Alcama immunohistochemistry (green) showed five pouches (p1-p5) in wild-type fish at 34 hpf. *tbx1*^−/−^ mutants lost all pouches except for the first (p1), whereas individual *nkx2.5*:Wnt11r or *nkx2.5*:Fgf8a-GFP transgenes modestly rescued, and combined *nkx2.5*:Wnt11r and *nkx2.5*:Fgf8a-GFP transgenes strongly rescued posterior pouches (p2-p5). Sensory ganglia are indicated with red asterisks. (P-T) Ventral views of dissected facial cartilages. A bilateral set of five CBs formed in wild-type zebrafish, and no CBs formed in *tbx1* mutants. No rescue of CB cartilage was seen in *nkx2.5*:Wnt11r; *tbx1*^−/−^ (*n*=39), *nkx2.5*:Fgf8a-GFP; *tbx1*^−/−^ (*n*=34), or *nkx2.5*:Wnt11r; *nkx2.5*:Fgf8a-GFP; *tbx1*^−/−^ larvae (*n*=31). (U) Quantification of pouch defects based on Alcama staining in wild type (*n*=51), *nkx2.5*:Wnt11r (*n*=49), *nkx2.5*:Fgf8a-GFP (*n*=36), *tbx1*^−/−^ (*n*=62), *nkx2.5*:Wnt11r; *tbx1*^−/−^ (*n*=44), *nkx2.5*:Fgf8a-GFP; *tbx1*^−/−^ (*n*=37), and *nkx2.5*:Wnt11r; *nkx2.5*:Fgf8a-GFP; *tbx1*^−/−^ (*n*=24). Data represent mean±s.e.m., *P* values are shown. (V) Low magnification view of an embryo at 32 hpf showing *nkx2.5*:GFP-positive mesoderm (green) relative to *her5*:mCherryCAAX-positive pouch endoderm (red) and the developing eye and ear. The schematic shows expression of *fgf8a* (green) and *wnt11r* (yellow) within distinct subsets of *nkx2.5*-positive mesoderm (red) during the formation of endodermal pouches (blue). Scale bars: 40 μm (A-O).

Given the crucial role for mesodermal Tbx1 in pouch formation, we next investigated its potential targets in this tissue. As the pouch defects of *tbx1* mutants are reminiscent of those seen upon inhibition of Wnt (Choe et al., 2013) or Fgf (Crump et al., 2004a) signaling, we explored whether the expression of individual Wnt and Fgf ligands might require Tbx1 function. In particular, *fgf8a* and *wnt11r* are expressed in the mesoderm during pouch initiation (Choe et al., 2013; Nechiporuk et al., 2007; Reifers et al., 2000), and we have previously reported requirements for both these genes in pouch morphogenesis (Choe et al., 2013; Crump et al., 2004a). In wild type, we observed expression of *fgf8a* and *wnt11r* in distinct subsets of the *nkx2.5*:GFP-positive mesoderm adjacent to pouch-forming endoderm (Fig. 3A,F,V). *wnt11r* appeared in the anterior arch mesoderm slightly before *fgf8a*, with expression of both shifting to more posterior mesoderm over time – consistent with the anterior to posterior progression of pouch formation (supplementary material Fig. S3A,B,E,F). This mesodermal expression of *fgf8a* and *wnt11r* was lost in *tbx1* mutants, and reintroduction of Tbx1 in *nkx2.5*-positive mesoderm (but not *nkx2.3*-positive endoderm) restored *fgf8a* and *wnt11r* expression (Fig. 3A-D,F-I). Also consistent with Wnt11r and Fgf8a acting downstream of Tbx1, we found that *tbx1* expression was unaltered in *wnt11r* or *fgf8a* mutants (supplementary material Fig. S3D,H). By contrast, expression of *wnt4a* in the overlying ectoderm, where it regulates the later maturation of pouch epithelia (Choe et al., 2013), as well as its endodermal receptor *fzd8a*, was largely unaffected in *tbx1* mutants (supplementary material Fig. S3L,N). The abnormal shape of the *wnt4a* ectodermal expression domain in *tbx1* mutants might reflect a requirement of endodermal contacts for proper expression, as *wnt4a* expression was lost in *sox32* mutants that lack endoderm (supplementary material Fig. S3M). Hence, abnormal *wnt4a* expression in *tbx1* mutants could be an indirect consequence of defective pouch outpocketing and altered endoderm-ectoderm contacts. In summary, we find that Tbx1 has a mesoderm-autonomous requirement for *fgf8a* and *wnt11r* expression, with the related *fgf3* and *wnt4a* ligands, as well as the *fzd8a* receptor, being regulated independently.

### Restoration of Wnt11r and Fgf8a in mesoderm rescues *tbx1^−/−^* pouch defects

As we found *wnt11r* and *fgf8a* to be positively regulated by Tbx1, we next tested whether restoring these proteins in mesoderm would rescue *tbx1* mutant pouches. To do so, we used the *nkx2.5* promoter to stably reintroduce Wnt11r and/or a green fluorescent protein (GFP)-tagged functional Fgf8a protein (Yu et al., 2009) into the *tbx1* mutant mesoderm, which we confirmed using *in situ* hybridization (Fig. 3E,J). In contrast to stably transgenic *nkx2.5*:Wnt11r embryos, which never displayed pouch or cartilage defects on their own, a few *nkx2.5*:Fgf8a-GFP embryos did display subtle pouch and ceratobranchial cartilage defects, consistent with the local guidance function of Fgf8a that is discussed below (supplementary material Fig. S3O-T). Compared with non-transgenic *tbx1*^−/−^ siblings, mutants expressing *nkx2.5*:Wnt11r or *nkx2.5*:Fgf8a-GFP alone formed a slightly greater number of pouches, with co-expression of both transgenes rescuing pouch number to a greater extent (Fig. 3K-O,U). As with mesodermal restoration of Tbx1, mesodermal restoration of Wnt11r, Fgf8a, or both failed to rescue ceratobranchial cartilage defects despite the rescue of pouches (Fig. 3P-T). Taken together, these findings support Wnt11r and Fgf8a as being major mesodermal effectors of Tbx1 for pouch morphogenesis.

### Synergistic effects of *wnt11r* and *fgf8a* loss on pouch formation

**Fig. 4.**
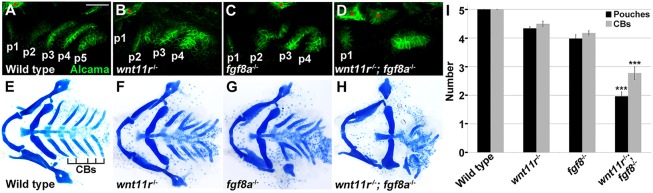
**Synergistic pouch and cartilage defects in compound *wnt11r*; *fgf8a* mutants.** (A-D) Alcama immunohistochemistry (green) labeled five pouches (p1-p5) in wild-type embryos at 34 hpf. Compared with the mild reductions of pouches in *wnt11r* or *fgf8a* single mutants, compound *wnt11r*^−/−^; *fgf8a*^−/−^ mutants had a near complete loss of pouches. Sensory ganglia are indicated with red asterisks. (E-H) Dissections of facial cartilage at 5 dpf. Wild type had five CBs on each side, *wnt11r* and *fgf8a* single mutants had variable fusions and losses of on average one CB and compound *wnt11r*^−/−^; *fgf8a*^−/−^ mutants lost multiple CBs. (I) Quantification of defects. Number of embryos examined (pouches, CBs): wild type (106, 102), *wnt11r*^−/−^ (66, 57), *fgf8a*^−/−^ (68, 98), and *wnt11r*^−/−^; *fgf8a*^−/−^ (24, 20). Data represent mean±s.e.m. ***P<0.001 relative to *fgf8a* mutants alone. Scale bar: 40 μm (A-D).

If Wnt11r and Fgf8a are parallel effectors of Tbx1 for pouch morphogenesis, then their combined loss should produce severe pouch defects that are similar to loss of *tbx1*. Individual *wnt11r* and *fgf8a* mutants form on average four pouches by 34 hpf and four ceratobranchial cartilages, compared with the normal complement of five each in wild-type zebrafish (Fig. 4). By contrast, *wnt11r*; *fgf8a* double mutants formed on average only two pouches and two to three ceratobranchials (Fig. 4D,H,I), with loss of just one copy of *wnt11r* enhancing the pouch and ceratobranchial defects of *fgf8a* mutants (supplementary material Fig. S4D,J,M). By contrast, loss of *wnt4a*, whose expression does not depend on Tbx1, failed to enhance the pouch and cartilage defects of *fgf8a* mutants (supplementary material Fig. S4F,L,M). Also consistent with parallel roles of Wnt11r and Fgf8a, we found that the mesodermal expression of each does not depend on the other (supplementary material Fig. S4N-Q).

### Reduced directional persistence of endodermal epithelial cells in *fgf8a* mutants

**Fig. 5.**
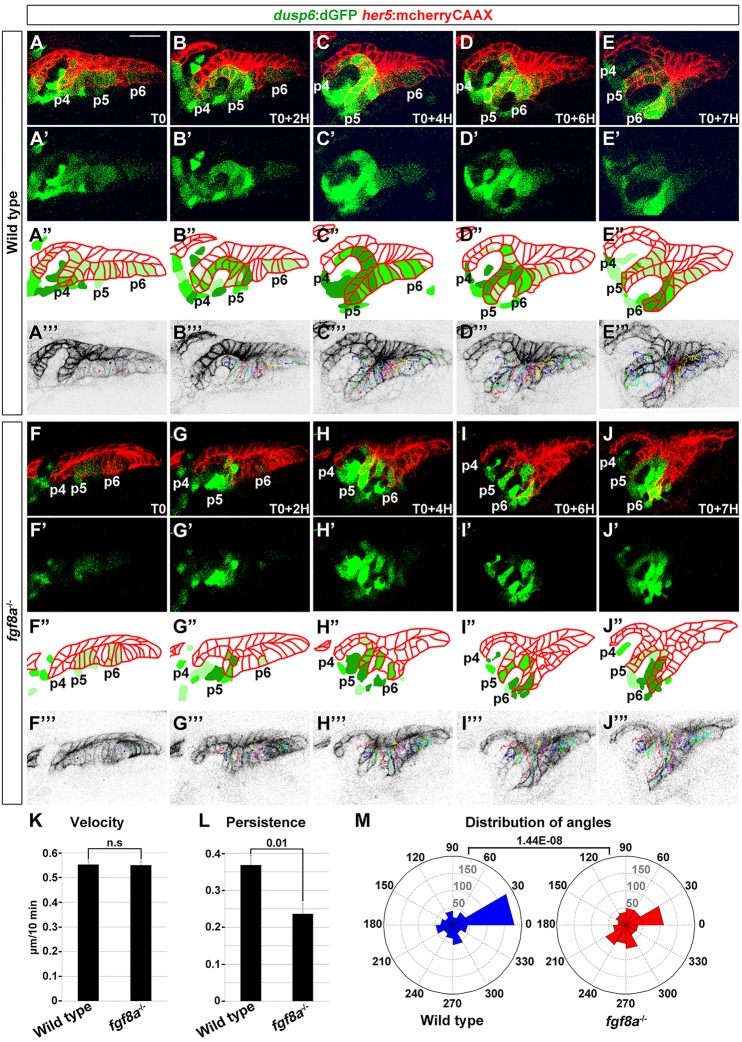
**Requirement for Fgf8a in the directional persistence of pouch cells.** (A-J) Representative confocal sections from time-lapse recordings show the development of pouches p4-p6 in a wild type (*n*=5) and *fgf8a* mutant (*n*=3) (see supplementary material Movie 3A,B). *her5*:mCherryCAAX labels endodermal cell membranes (red) and *dusp6*:dGFP shows dynamic Fgf activity (green). T0 is 26 hpf. Merged images are shown in A-J and *dusp6*:dGFP alone in A″-J″. Schematics in A′-J′ show the graded intensity of *dusp6*:dGFP (green) relative to endodermal cells (red). Individual cell tracks used for the quantification are shown in A‴-J‴. (K-M) The velocity, persistence of migration and distribution of angles of tracked cells over a 7-h period. For distribution of angles, each bin represents the number of cells moving in a particular direction relative to the last cell position. We tracked the cells of two embryos for each wild type and *fgf8a* mutant. Mean±s.e.m. and *P* values are shown. n.s., not significant. Scale bar: 20 µm (A-J).

We have previously reported that Wnt11r functions to initially destabilize the pouch-forming endoderm so that these epithelial cells can initiate outpocketing (Choe et al., 2013). However, in contrast to *wnt11r* mutants in which the multi-layering of pouch-forming cells is delayed, we found that *fgf8a* mutant epithelial cells did become multi-layered at the initial stages (supplementary material Fig. S5G-I). Hence, we performed time-lapse recordings of pouch development, using *her5*:mCherryCAAX to track individual epithelial cells and *dusp6*:dGFP to monitor dynamic Fgf activity, to assess the potential later requirements for Fgf8a in epithelial cell behavior. In all five wild-type embryos (Fig. 5A-E; supplementary material Movies 2, 3A), we observed segmental clusters of *dusp6*:dGFP-positive cells in both forming pouches and presumptive pre-pouch zones, as well as in the adjacent mesoderm. By tracking individual epithelial cells, we found that those that initially expressed *dusp6*:dGFP or turned it on during the recording selectively contribute to growing pouches compared with *dusp6*:dGFP-negative cells, consistent with Fgf activity prefiguring the later contributions of cells to pouches (supplementary material Fig. 5). For *fgf8a* mutants, several differences were consistently observed in three separate embryos (Fig. 5F-J; supplementary material Movie 3B). First, fewer *her5*:mCherryCAAX-positive epithelial cells expressed *dusp6*:dGFP in the pre-pouch endoderm and did so at lower levels; mesodermal *dusp6*:dGFP expression was affected to a lesser extent. Second, nascent mutant pouches comprised mosaics of cells with high, low, or no *dusp6*:dGFP, compared with the more uniform high expression levels of *dusp6*:dGFP in wild-type pouches. By tracking individual pouch-forming cells in wild-type and *fgf8a* mutant zebrafish, we quantified how loss of Fgf8a affects the migratory behavior of these epithelial cells. Although wild-type and *fgf8a^−/−^* cells had similar velocities, we found that *fgf8a^−/−^* cells had less-directed migration paths, as measured by both the persistence of migration and the distribution of the angles of migration (Fig. 5K-M). One interpretation of this is that the mosaic reduction of endodermal Fgf activity results in reduced directional coherence of migrating pouch cells, which in turn might explain the wide variation of pouch defects that has been observed in *fgf8a* mutants (Crump et al., 2004a).

### Deviation of growing pouches by ectopic Fgf8a requires Wnt11r function

**Fig. 6.**
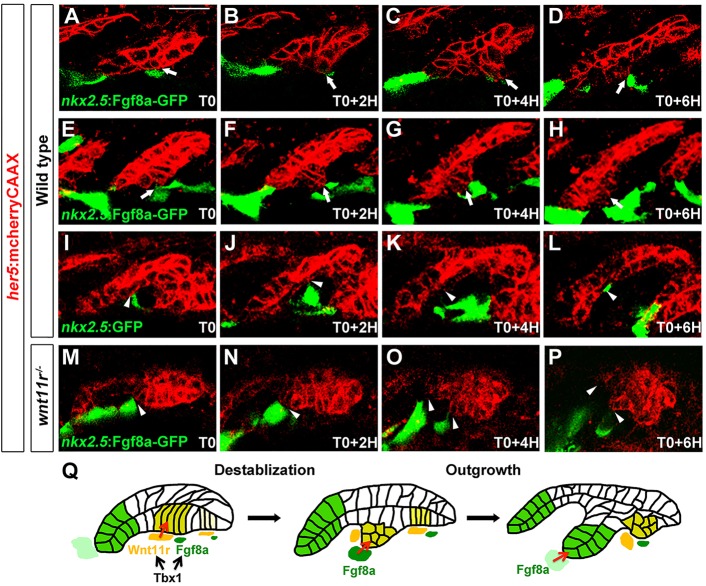
**Ectopic Fgf8a redirects pouch outgrowth.** (A-P) Still images from time-lapse recordings monitoring the development of the third pouch in wild-type embryos with mosaic mesodermal mis-expression of a *nkx2.5*:Fgf8a-GFP transgene (two examples are shown: A-D and E-H) or a control *nkx2.5*:GFP transgene (I-L), as well as *wnt11r* mutants with *nkx2.5*:Fgf8a-GFP mis-expression (M-P) (see supplementary material Movie 4). *her5*:mCherryCAAX labels endodermal cell membranes in red. Recordings started at 25 hpf (T0), and subsequent stills were taken every 2 h. *nkx2.5*:Fgf8a-GFP-expressing clones transiently diverted developing third pouch cells (arrows) in four out of six embryos, whereas *nkx2.5*:GFP-expressing clones had no effect on adjacent third pouch cells (arrowheads) in all three of the embryos examined. *nkx2.5*:Fgf8a-GFP-expressing clones failed to attract *wnt11r^−/−^* endodermal cells (arrowheads) in all embryos (*n*=3). (Q) A two-step model of Tbx1 function in pouch morphogenesis. Tbx1 promotes *wnt11r* and *fgf8a* expression in distinct domains of the mesoderm, with Wnt11r initiating pouch morphogenesis through epithelial destabilization and Fgf8a guiding subsequent pouch outgrowth. Scale bar: 20 μm (A-P).

The analysis of *dusp6*:dGFP in *fgf8a* mutants indicates a transcriptional response to Fgf signaling in the endoderm that, at least in part, requires mesodermal Fgf8a. In order to investigate whether Fgf8a might also have more immediate effects on directed pouch outgrowth, we used time-lapse imaging to assess the effects of ectopic Fgf8a-GFP mesodermal clones on neighboring pouch epithelial cells. To do so, we took advantage of our observation that expression of the endogenous *fgf8a* gene was restricted to a ventral subset of the *nkx2.5*-positive mesoderm (Fig. 3A), and that injection of DNA into one-cell-stage zebrafish embryos results in mosaic transgene expression during later development. We therefore injected the *nkx2.5*:Fgf8a-GFP transgene into *her5*:mCherryCAAX embryos and selected those pouch-stage embryos with clones of Fgf8a-GFP in more dorsal mesoderm. In four out of six wild-type embryos, time-lapse imaging revealed that ectopic expression of Fgf8a-GFP in dorsal mesodermal cells caused adjacent pouch-forming epithelial cells to diverge from their normal lateral migration path (two examples are shown in Fig. 6A-H; supplementary material Movie 4A,B). Eventually, diverted pouch-forming cells resumed their normal lateral migration path, perhaps in response to the endogenous *fgf8a*-expressing mesodermal cells that were located more ventrally. As a control, we observed no effects on pouch outgrowth in all three wild-type embryos that expressed a *nkx2.5*:GFP transgene in similar domains of dorsal mesoderm (Fig. 6I-L; supplementary material Movie 4C).

Next, we investigated what role the Wnt11r-dependent destabilization of the pre-pouch epithelium plays in the ability of cells to respond to ectopic Fgf8a. Consistent with Wnt11r being required for the Fgf8a response, ectopic *nkx2.5*:Fgf8a-GFP mesodermal clones failed to attract adjacent presumptive pouch epithelial cells in all three *wnt11r* mutants examined (Fig. 6M-P; supplementary material Movie 4D). This inability of *wnt11r^−/−^* epithelial cells to efficiently respond to Fgf8a was not due to a requirement for Wnt11r in the Fgf transcriptional response, as *dusp6*:dGFP expression was unaffected in *wnt11r* mutants (supplementary material Fig. S6). Instead, our data are consistent with the notion that Wnt11r-mediated destabilization of the pre-pouch epithelium is required for cells to be guided by local sources of Fgf8a in the ventral *nkx2.5*-positive mesoderm (Fig. 6Q).

## DISCUSSION

Our study reveals that Tbx1 functions in the facial mesoderm to coordinate multiple steps in the outpocketing of the endodermal epithelium into pouches. Through high-resolution recordings of pouch development and transgenic rescue experiments, we provide evidence that Tbx1 initiates epithelial morphogenesis through Wnt11r and then guides directional pouch outgrowth through Fgf8a. This previously underappreciated morphogenetic role of mesodermal Tbx1 in pouch development helps to clarify the tissue-specific functions and molecular targets of this crucial DiGeorge syndrome gene during craniofacial development.

### Morphogenetic role of mesodermal Tbx1 for endodermal pouch development

Our rescue experiments using the *nkx2.5*:Tbx1 transgene establish important roles of Tbx1 within the facial mesoderm for the outpocketing of the endoderm into pouch epithelia. Restoration of Tbx1 to the *nkx2.3*-positive endoderm failed to rescue pouch development on its own, but it did improve the extent of pouch rescue upon expression of *nkx2.5*:Tbx1. Thus, Tbx1 probably functions in both the mesoderm and endoderm for pouch development. The observed enhanced rescue by the *nkx2.5*:Tbx1 transgene alone, compared with the *nkx2.3*:Tbx1 transgene, might reflect a more prominent role of mesodermal, compared with endodermal, Tbx1 for pouch formation. Alternatively, the *nkx2.3*:Tbx1 transgene might not restore Tbx1 early enough or to sufficient levels in the endoderm to strongly rescue pouches. Indeed, *nkx2.3*:Tbx1 restored *tbx1* expression primarily to just the posterior pouch-forming endoderm.

A mesodermal role for Tbx1 in zebrafish is consistent with mouse studies showing that tissue-specific deletion of *Tbx1* in the *Mesp1*-positive mesoderm results in severe pouch defects and that restoration of *Tbx1* in *Mesp1*-positive mesoderm largely rescues pouch defects in *Tbx1* null embryos (Zhang et al., 2006). We used a mesoderm-specific *nkx2.5* promoter to restore *tbx1* and rescue pouches, but deletion of *Tbx1* with an *Nkx2.5*:Cre driver in mouse has no effect on pouch development (Xu et al., 2004). This discrepancy might be explained by the inefficient and hence mosaic activity of this *Nkx2.5*:Cre line (Zhang et al., 2005), or by different timings of *Nkx2.5* gene expression between mouse and fish. In mouse, deletion of *Tbx1* using an endoderm-specific isolate of the *Foxg1*:Cre construct also resulted in pouch defects, and endoderm-specific *Tbx1* deletion with *Fgf15*:Cre resulted in loss of the fourth pharyngeal artery, which might be interpreted as secondary to altered development of the fourth pouch (Arnold et al., 2006; Zhang et al., 2005). In mouse, endodermal Tbx1 has been proposed to promote the proliferation of pouch endodermal progenitors (Arnold et al., 2006; Xu et al., 2005). One explanation then for the pouch defects that are seen upon endoderm-specific *Tbx1* deletion in mouse is that there are simply less cells available to make normal pouches. Indeed, we also observed extensive proliferation in the pre-pouch endoderm of zebrafish, although only limited proliferation was apparent during pouch outpocketing (data not shown). Hence, a parsimonious view is that Tbx1 acts primarily in the mesoderm for pouch morphogenesis yet has additional roles in the endoderm for other aspects of pouch cell biology, such as their proliferative expansion.

### Segmental patterning of the pre-pouch endoderm in the absence of Tbx1

Tbx1 is essential for the morphological outpocketing of the endoderm into pouches; however, we find that the pre-pouch endoderm still retains some segmental characteristics even in the absence of Tbx1. In particular, expression of the *fgf3* ligand and Fgf activity itself remain apparent in segmental clusters of *tbx1* mutant endodermal cells despite their failure to undergo morphogenesis. Consistent with Tbx1 being dispensable for the initial segmentation of pharyngeal endoderm, hemichordates segment an apparently homologous population of pharyngeal endoderm despite lacking *Tbx1* expression in their pharynx (Gillis et al., 2012). One explanation is that pharyngeal segmentation evolutionarily pre-dates *Tbx1* expression, with mesodermal Tbx1 in vertebrates driving more complex morphologies of pouches, coinciding with the newfound investment of pharyngeal arches with neural crest cells. Although the nature of this early Tbx1-independent segmentation of the endoderm remains unclear, it is interesting that the Fgf reporter *dusp6*:dGFP is an early marker of epithelial cells that are destined to generate pouches. In the pre-somitic mesoderm, iterative Fgf activity plays an important role in the segmentation of this tissue into somites (reviewed by Pourquié, 2011). Hence, it will be interesting to explore whether the pharyngeal endoderm utilizes a similarly constructed segmentation clock.

### Wnt11r is a novel morphogenetic effector of Tbx1 in pouch development

Several lines of evidence indicate that *wnt11r* is a crucial effector of Tbx1 for pouch development. First, time-lapse imaging of *tbx1* mutants revealed a failure to initiate outpocketing of the endodermal epithelium, a phenotype similar to that of *wnt11r* mutants and embryos in which the cytoplasmic Wnt effectors Dishevelled and Rac1 have been inhibited (Choe et al., 2013). Second, *wnt11r* expression was lost in the *nkx2.5*-positive mesoderm of *tbx1* mutants. Third, restoration of Wnt11r in this mesoderm partially rescued *tbx1^−/−^* pouch defects, and did so more effectively when combined with Fgf8a. Recently, a role for Wnt11r downstream of Tbx1 has been reported for the looping of the heart in zebrafish (Choudhry and Trede, 2013). This dual requirement of Tbx1 in the outpocketing of pouch endoderm and looping of heart mesoderm might reflect a common regulation of *wnt11r* in the *nkx2.5*-positive mesoderm, with this mesodermal subpopulation not only contributing to the myocardium but also organizing the adjacent pouch endoderm. In mice, the *wnt11r* homolog *Wnt11* has a similar requirement to *Tbx1* for development of the second heart field, although this is only revealed when *Wnt5a* is also disrupted (Cohen et al., 2012). *Wnt11^−/−^* mutants are viable and presumably lack the pouch defects seen in *wnt11r* mutant fish, but pouch development in *Wnt11*; *Wnt5a* double mutants, which die by embryonic day (E)10.5, has not been analyzed. Indeed, the relatively weaker pouch defects of *wnt11r* mutants versus embryos expressing dominant-negative Wnt signaling constructs indicates the presence of other functionally redundant Wnt proteins downstream of Tbx1 in pouch development (Choe et al., 2013).

### Guidance role of Fgf signaling in pouch epithelial outpocketing

The ability of mesodermal Fgf8a plus Wnt11r to rescue *tbx1* mutant pouches indicates an important role for mesoderm-derived Fgf ligands in Tbx1-dependent pouch morphogenesis. Our results are consistent with those in mouse that show loss of mesodermal *Fgf8a* expression upon deletion of *Tbx1* in the *Mesp1*-positive mesoderm (Zhang et al., 2006). Although it has been reported that insertion of *Fgf8* into the *Tbx1* locus failed to rescue *Tbx1* mutant defects (Vitelli et al., 2006), this could be due to ectopic expression of *Fgf8* in non-mesodermal *Tbx1*-positive tissues or the need to also restore Wnt ligands. It has been proposed that Fgf8 in mouse functions in a tissue-autonomous manner for mesodermal cell proliferation (Zhang et al., 2006). By contrast, our finding that expression of the Fgf reporter *dusp6*:dGFP is reduced in the endoderm but not mesoderm of zebrafish *fgf8a* mutants suggests that there is a non-autonomous requirement for mesodermal Fgf8a in endoderm development.

Cellular resolution time-lapse imaging also provides direct evidence for a role of Fgf8a in guiding collective cell migration during pouch outpocketing. Guidance roles for Fgf signaling have been well characterized in the *Drosophila* tracheal system (Ghabrial and Krasnow, 2006; Sutherland et al., 1996) and recently in the migration of *Drosophila* caudal visceral mesoderm cells (Kadam et al., 2012). In vertebrates, Fgf10 promotes the collective migration of posterior lateral line neuroblasts in zebrafish (Breau et al., 2012), whereas Fgf4 and Fgf8 appear to oppositely attract and repel mesenchymal cells of the chick primitive streak (Yang et al., 2002). Although *Fgf10* is essential for branching of the endodermal epithelium to form the mammalian lung, a direct role in guiding collective epithelial cell migration has not been provided (Bellusci et al., 1997; Sekine et al., 1999). Our finding that ectopic sources of Fgf8a can redirect pouch epithelial migration therefore raises the possibility that Fgf ligands might guide the collective migration of branching epithelia along the length of the endoderm.

The observation that Fgf8a is required for a transcriptional response in the endoderm (i.e. expression of *dusp6*:dGFP) might seem at odds with the chemotactic role that we have revealed using ectopic Fgf8a-GFP mis-expression, with chemotaxis presumably involving rapid cytoskeletal changes in the cytoplasm. By contrast, work in the lateral line system has shown that Fgf signaling can influence collective cell migration by controlling chemokine receptor expression (Aman and Piotrowski, 2008; Nechiporuk and Raible, 2008). Previous studies have also shown that Fgf signaling can elicit multiple responses within a cell through distinct downstream pathways (Baron et al., 2000; Maher, 1999; Raucci et al., 2004), and hence Fgf signaling in the growing pouch might also serve to coordinate chemotactic cytoskeletal changes with nuclear transcription responses that promote the maturation, proliferation and/or survival of pouch cells. Further work is also needed to elucidate how Wnt11r induces competency of the endodermal epithelium to respond to Fgf8a. One possibility is that Wnt11r-dependent destabilization of adherens junctions allows pre-pouch epithelial cells to more easily extend membrane extensions that promote migration towards Fgf8a sources. The cytoplasmic effectors of Fgf8a signaling that promote chemotaxis remain unknown, but it is possible that Wnt11r and Fgf8a signaling might converge on common targets in the cytoplasm that promote collective cell migration. By contrast, Wnt11r does not appear to be required for either *fgf8a* expression or the Fgf transcriptional response, supporting our earlier work that cytoplasmic and not nuclear Wnt signaling mediates pouch morphogenesis (Choe et al., 2013).

### Pouch-independent roles for Tbx1 in craniofacial skeletal development

A surprising finding from our study was the lack of craniofacial skeletal rescue despite the robust rescue of pouch morphogenesis in *tbx1* mutants. This was true when Tbx1 was restored to both the mesoderm and endoderm of *tbx1* mutants, or when combined Wnt11r and Fgf8a were restored to the mesoderm. It is well established that defective pouch development results in decreased cartilage and bone formation from the hyoid and more posterior branchial arches, with the latter generating the ceratobranchial cartilages that are most affected by the loss of *tbx1* in zebrafish. For example, mutations in either *integrinα5* or *sox32* in zebrafish result in both pouch defects and losses of the posterior facial skeleton, with skeletal rescue by wild-type endoderm transplants demonstrating that skeletal defects are due to non-autonomous roles of these genes in the endoderm (Crump et al., 2004b; David et al., 2002). Our data indicate that, beyond its role in inducing pouch formation, Tbx1 also has an earlier role in the development of the skeletogenic neural crest. In particular, we observed reduced expression of the ectomesenchyme marker *dlx2a* in the posterior arches of *tbx1* mutants, even at stages before those at which the pouches normally form. At least in mouse, Tbx1 does not appear to function cell-autonomously within cranial neural crest cells to promote their ectomesenchyme potential, as neural-crest-specific deletion of *Tbx1* using the *Wnt1*:Cre driver does not result in any craniofacial defects (Aggarwal et al., 2010). Instead, it is likely that Tbx1 has non-autonomous requirements for early cranial neural crest development, for example within mesoderm and/or endoderm populations that are not labeled by the *nkx2.5* and *nkx2.3* promoters used in this study. Indeed, the dissociation of pouch and craniofacial skeletal defects in these ‘rescued’ *tbx1* mutants further highlights the multiplicity of functions of this transcription factor in the assembly of the vertebrate head.

## MATERIALS AND METHODS

### Zebraﬁsh lines

Zebrafish (*Danio rerio*) were raised in strict accordance with good animal practice as defined by the relevant national and/or local animal welfare bodies, and all animal work was approved by the University of Southern California Institutional Animal Care and Use Committee. Published lines used include *tbx1^tu285^* (*van gogh*) (Piotrowski et al., 2003), *wnt11r^fh224^* (Banerjee et al., 2011), *fgf8a^ti282a^* (*acerebellar*) (Reifers et al., 1998), *Tg*(∼*3.4her5:EGFP*)*^ne1911^* (Tallafuss and Bally-Cuif, 2003), *Tg*(*her5:mCherryCAAX*)*^el72^* (Choe et al., 2013), *Tg*(*nkx2.5:GFP*)*^el83^* (Witzel et al., 2012), *Tg*(*sox10:LOX-GFP-LOX-hDLX3*)*^el8^* (Das and Crump, 2012), and *Tg*(*dusp6:dGFP*) (Molina et al., 2007). For genotyping of *tbx1*^tu*285*^, primers TBX-09 and TBX-10 converted *tbx1^tu285^* into a co-dominant polymorphism, with a wild-type product of 438 bp and mutant products of 276 and 162 bp after *Pac*I digestion. Genotyping of *wnt11r^fh224^* was as described previously (Banerjee et al., 2011). *fgf8a^ti282a^* mutant embryos were scored by loss of cerebellum (Reifers et al., 1998). *nkx2.3*:Tbx1, *nkx2.5*:Tbx1, *nkx2.5*:Wnt11r and *nkx2.5*:Fgf8a-GFP transgenic constructs were generated using the Gateway (Invitrogen) Tol2kit (Kwan et al., 2007). p5E-*nkx2.3*, p5E-*nkx2.5* and pME-Wnt11r plasmids have been published previously (Choe et al., 2013). For pME-Tbx1, the coding sequence of *tbx1* was amplified using primers Tbx1-B1F and Tbx1-B2R from multi-stage zebrafish cDNA according to the manufacturer’s instructions (Phusion, NEB) and cloned into pDONR221. The Fgf8a-GFP fusion protein was generated through fusion PCR (Szewczyk et al., 2007) to insert GFP between amino acid residues 22 and 23 of Fgf8a. This fusion has been previously verified to be functional (Yu et al., 2009). The Fgf8a-GFP cDNA was then cloned into pDONR221 to generate pME-Fgf8a-GFP. After LR cloning with p3E-pA and pDestTol2CG2 (*cmlc2*:GFP) or pDestTol2AB (*α-crystallin*:Cerulean), plasmids were injected at 30 ng/μl with 35 ng/μl Tol2 transposase RNA into one-cell stage embryos. Four independent transgenic lines were isolated for *Tg*(*nkx2.3:Tbx1:pA*) and *Tg*(*nkx2.5:Wnt11r:pA*) based on *cmlc2*:GFP heart fluorescence. Two independent transgenic lines were isolated for *Tg*(*nkx2.5:Tbx1:pA*) based on *α-crystallin*:Cerulean lens fluorescence. Four independent transgenic lines were isolated for *Tg*(*nkx2.5:Fgf8a-GFP:pA*) based on mesodermal GFP fluorescence. We used stable lines *Tg*(*nkx2.3:Tbx1:pA*)*^el513^*, *Tg*(*nkx2.5:Tbx1:pA*)*^el567^*, *Tg*(*nkx2.5:Wnt11r:pA*)*^el493^* and *Tg*(*nkx2.5:Fgf8a-GFP:pA*)*^el562^* for this study. In order to generate *nkx2.5*:Fgf8a-GFP mosaics, 30 ng/μl of the *nkx2.5*:Fgf8a-GFP construct with 35 ng/μl Tol2 RNA was injected into one-cell stage wild-type or *wnt11r* mutant *her5*:mCherryCAAX embryos. We then used a Leica M165 FC fluorescence dissecting stereoscope with 3.2× magnification to select mosaic animals displaying desired GFP fluorescence at 22 hpf, use of confocal microscopy allowed further selection for time-lapse imaging. See supplementary Materials and Methods for primers.

### Staining

Immunohistochemistry for Alcama/ZN8 (Zebrafish International Resource Center, 1:400) was performed as described previously (Crump et al., 2004a). Alcian Blue staining, fluorescent *in situ* hybridizations and GFP immunohistochemistry (Torrey Pines Biolabs, 1:1000) protocols have been published previously (Zuniga et al., 2011). The *in situ* probes included *wnt11r*, *wnt4a* and *fzd8a* (Choe et al., 2013). For *tbx1*, *fgf8a* and *fgf3*, probes were generated by using PCR and cloned into pGEM^®^-T Easy Vector Systems (Promega), and digoxigenin-labeled RNAs were synthesized using T7 or SP6 RNA polymerase. See supplementary Materials and Methods for primers. Direct visualization of LysoTracker Red staining was used to monitor cell death in embryos and antibody-mediated staining of BrdU incorporation was used to follow cell division. Further details are given in the supplementary Materials and Methods.

### Imaging

Craniofacial cartilages were dissected with fine insect pins and were then flat-mounted for imaging on a Leica DM 2500 upright microscope using Leica software. Fluorescence images of antibody-stained or *in situ* hybridization embryos were taken on a Zeiss LSM5 confocal microscope using ZEN software. For time-lapse imaging, live embryos were mounted as previously described (Crump et al., 2004a), and approximately 80-μm *z*-stacks at 1.5-μm intervals were captured by using a Zeiss 40× LD-Plan Neofluar objective lens every 10 min. For single section movies, time-lapse imaging datasets were manually assembled using ZEN software, and then the brightness and contrast of movies were adjusted using Fiji. For cell tracking, we used Fiji freeware to manually annotate the centroid of each pouch cell in each frame of the time-lapse recording. Only cells that could be reliably tracked throughout the whole 6 h window were used for analysis. Cell tracks were then exported to MATLAB (MathWorks) and processed as described previously (Matthews et al., 2008). Velocity was calculated as the total distance traveled divided by time, and persistence equals the ratio between the linear distance from the initial to the final point and the total length of the migratory path. For the angle of migration, lines were first generated between the first and second track position and then compared with the lines generated between the second and third track position. The angle represents the deviation between these lines. The process was then performed iteratively for each subsequent set of time points (i.e. the line between third and fourth track compared with the line between second and third track) and for each cell in the analysis.

### Scoring and statistics

Pouches missing or reduced >50% compared with those of wild type were scored as 0 and mis-shapen or normal pouches as 1 (examples shown for each are shown in supplementary material Fig. S7). Wild-type zebrafish invariantly had five pouches per side at 34 hpf. We scored fusions of two ceratobranchials as 1.5, normal ceratobranchials as 1.0, reduced ceratobranchials as 0.5 and absent ceratobranchials as 0. We utilized the multiple comparison test of Tukey–Kramer for pouch and ceratobranchial defects, one-tailed Student's *t*-test with unequal variance for cell velocity and persistence, chi-square test of independence for distribution of angles and Fisher's exact test for contributions of *dusp6*:dGFP-positive cells to pouches.

## Supplementary Material

Supplementary Material
