# Supplementary material for: Tbx1 controls the morphogenesis of pharyngeal pouch epithelia through mesodermal Wnt11r and Fgf8a
Source: Development. 2014 Sep;141(18):3583–93. doi: 10.1242/dev.111740 (PMC4197720; doi:10.1242/dev.111740)
Supplement: Supplementary Material [file supp_141_18_3583__index.html]

Tbx1 controls the morphogenesis of pharyngeal pouch epithelia through mesodermal Wnt11r and Fgf8a — Supplementary Material 

# Tbx1 controls the morphogenesis of pharyngeal pouch epithelia through mesodermal Wnt11r and Fgf8a

## DEV111740 Supplementary Material

**Files in this Data Supplement:**

- **Supplementary Material**
