# Supplementary material for: Tbx1 controls the morphogenesis of pharyngeal pouch epithelia through mesodermal Wnt11r and Fgf8a
Source: Development. 2014 Sep;141(18):3583–93. doi: 10.1242/dev.111740 (PMC4197720; doi:10.1242/dev.111740)
Supplement: Supplementary Material [file supp_141.18.3583_DEV111740.pdf]

SUPPLEMENTARY MATERIALS AND METHODS

BrdU and Lysotracker staining

Live embryos were manually removed from their chorions and incubated in darkness with BrdU staining solution for 30 minutes or Lysotracker Red solution for 45 minutes. BrdU staining solution consisted of 15 mM of BrdU (Sigma) in 15% DMSO / embryo media. Lysotracker Red solution consisted of Lysotracker Red (Invitrogen) diluted to 5 μM in embryo media. After 4–5 rinses with embryo media, embryos were fixed overnight with 4% PFA in PBS at 4°C. Embryos were then rinsed twice with PBT before dehydration in a methanol/PBT series to remove background, and then rehydrated and washed twice with PBT. BrdU embryos were processed for immunohistochemistry with a rat monoclonal anti-BrdU antibody (1:200, BioRad) and secondary antibody Alexa Fluor 647 Goat Anti-Rat IgG (H+L) (1:200, Molecular Probes). Lysotracker Red embryos were directly imaged by confocal microscopy.

Genotyping primers

TBX_09: 5’-TCCAACTCAGCACAAGCCCC-3’
TBX_10: 5’- CCAATCAAGTGCATTGACGATG-3’

Primers used to create transgenic constructs

Tbx1-B1F: 5’-GGGGACAAGTTTGTACAAAAAAGCAGGCTCCACCATGATTTCAGCAATATCAAGC-3’
Tbx1-B2R: 5’-GGGGACCACTTTGTACAAGAAAGCTGGGTTTATCTGGGTCCGTAGTCAT-3’
Fgf8-GFP-F: 5’-ATTACAGGCGCATCAGAG-3’
Fgf8-GFP-R2: 5’-TGCTCACCATAGCATAGTAGC-3’
Fgf8-GFP-F2: 5’-GCTACTATGCTATGGTGAGCA-3’
Fgf8-GFP-R3: 5’-GGTTACCTGCTTGTACAGC-3’
Fgf8-GFP-F3: 5’-GCTGTACAAGCAGGTAACC-3’
Fgf8-GFP-R: 5’-TCAACGCTCTCCTGAGTAG-3’
Fgf8-GFP-B1F: 5’-GGGGACAAGTTTGTACAAAAAAGCAGGCTATTACAGGCGCATCAGAG-3’
Fgf8-GFP-B2R: 5’-GGGGACCACTTTGTACAAGAAAGCTGGGTTCAACGCTCTCCTGAGTAG-3’

Primers used to create in situ probes

tbx1-r-F: 5’-GGATCCATACAGCCATCA-3’
tbx1-r-R: 5’-GGATCCACGTAGACCACA-3’
fgf8a-r-F: 5’-CAGGTAACCATTCAGTCC-3’
fgf8a-r-R: 5’-TCAACGCTCTCCTGAGTAG-3’
fgf3-r-F: 5’-CCGAGTTTGGAGGAATCT-3’
fgf3-r-R: 5’-GTTTTAAAGCCCCTCCTG-3’

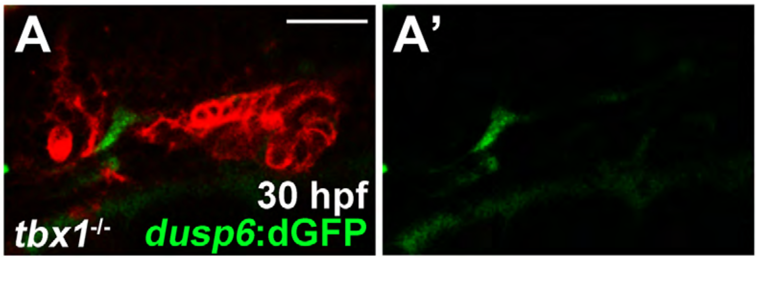

Fig. S1, Original image of *dusp6*:dGFP; *her5*:mCherryCAAX; *tbx1*$^{-/-}$ embryos
(A) The same image as Figure 1O but without increasing the gain. This image shows that overall *dusp6*:dGFP expression is very reduced in *tbx1* mutants compared to the wild-type control of Figure 1N. Scale bar = 40 μM.

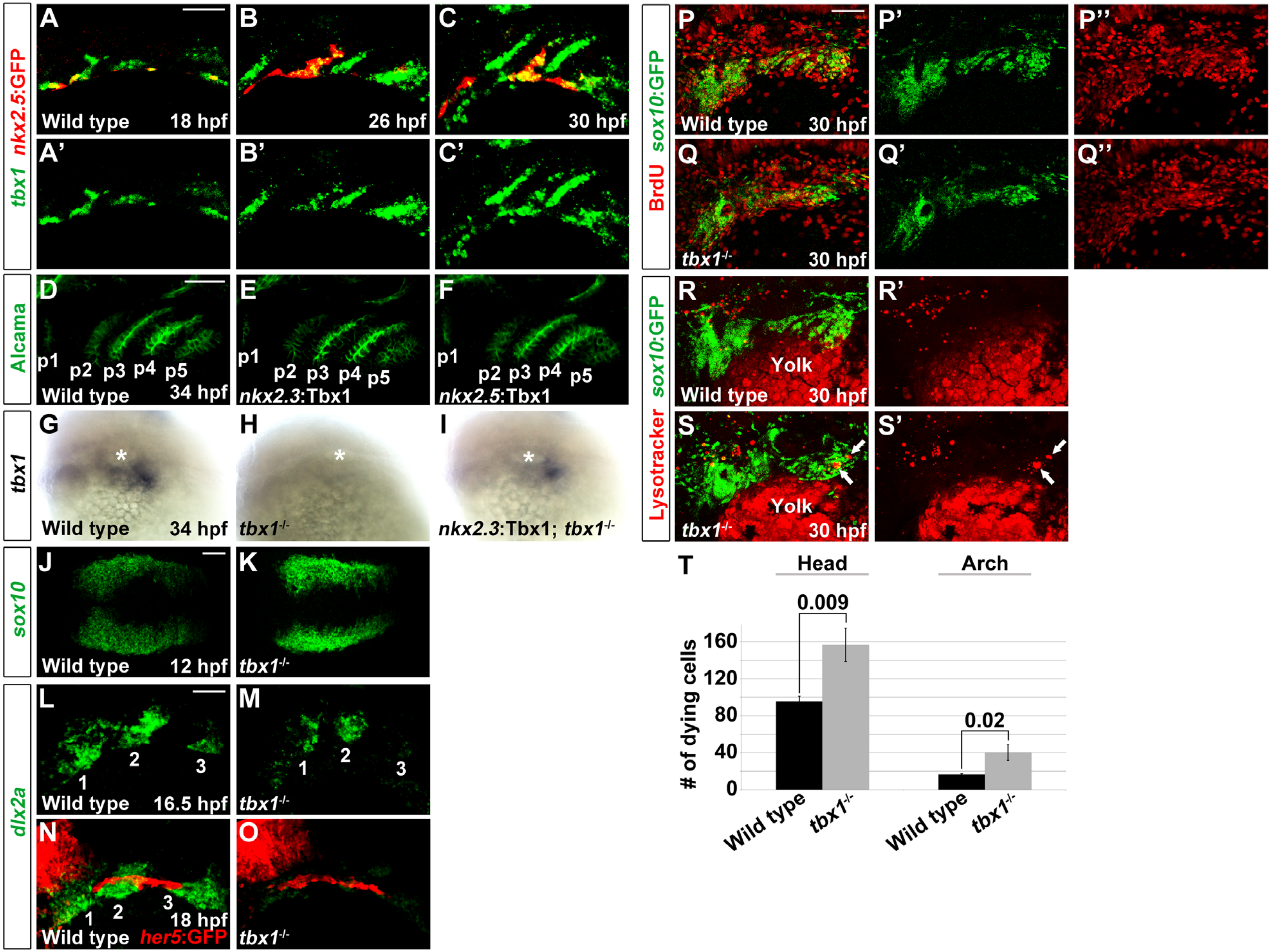

tbx1 nkx2.5:GFP
A
Wild type 18 hpf
B
26 hpf
C
30 hpf
A'
B'
C'
Alcama
D
p1 p2 p3 p4 p5
Wild type 34 hpf
E
p1 p2 p3 p4 p5
nkx2.3:Tbx1
F
p1 p2 p3 p4 p5
nkx2.5:Tbx1
tbx1
G
Wild type 34 hpf
H
tbx1-/-
I
nkx2.3:Tbx1; tbx1-/-
sox10
J
Wild type 12 hpf
K
tbx1-/-
dlx2a
L
1 2 3
Wild type 16.5 hpf
M
1 2 3
tbx1-/-
N
1 2 3
18 hpf
Wild type her5:GFP
O
tbx1-/-
BrdU sox10:GFP
P
Wild type 30 hpf
P'
P''
Q
tbx1-/- 30 hpf
Q'
Q''
Lysotracker sox10:GFP
R
Yolk
Wild type 30 hpf
R'
S
Yolk
tbx1-/- 30 hpf
S'
T
Head
Arch
0.009
0.02
# of dying cells
0
40
80
120
160
Wild type
tbx1-/-
Wild type
tbx1-/-

Fig. S2, Mesodermal *tbx1* expression during pouch formation and a requirement for Tbx1 in ectomesenchyme development
(A-C) Fluorescent in situ hybridization shows expression of *tbx1* (green) in *nkx2.5*:GFP+ mesoderm labeled by GFP immunohistochemistry (red), as well as in *nkx2.5*:GFP- endoderm. Anterior is to the left and ventral is down. Scale bar represents 20 μM.

(D-F) Alcama immunohistochemistry on 34 hpf embryos. Transgenic expression of Tbx1 in the endoderm or mesoderm of *nkx2.3*:Tbx1 or *nkx2.5*:Tbx1 embryos, respectively, does not affect pouch development.

(G-I) In situ hybridization for *tbx1* (dark blue) in 34 hpf embryos. In 31/31 wild-type siblings, *tbx1* expression is observed in the pharyngeal region ventral to the ear (asterisk). In 8/8 *tbx1* mutant siblings, all *tbx1* expression is lost. In 11/11 *nkx2.3*:Tbx1; *tbx1*$^{-/-}$ embryos, *tbx1* expression is partially restored to the pharyngeal region, particularly posterior to the ear.

(J and K) Fluorescent in situ hybridization for *sox10* (green) at 12 hpf. Dorsal views with anterior to the left show *sox10* expression in two bilateral fields of neural crest cells in both wild types and *tbx1* mutants. We also note that *sox10* expression was consistently higher in *tbx1* mutants.

(L-O) Fluorescent in situ hybridization for *dlx2a* (green) to detect neural-crest-derived ectomesenchyme. While *dlx2a* is expressed in three distinct neural crest streams in 11/11 wild-type siblings at 16.5 hpf, its expression is reduced in the first and second streams and nearly lost in the third stream in 3/5 *tbx1* mutants. Compared to wild-type siblings at 18 hpf (n=21), the arch expression of *dlx2a* was greatly reduced in 4/4 *tbx1* mutants. *her5*:GFP labeling of endoderm at 18 hpf (red) shows that pouches have yet to form at this stage. Scale bar represents 40 μM.

(P and Q) BrdU staining (red) labels mitotic cells, and *sox10*:GFP (green) labels neural-crest-derived ectomesenchyme cells of the pharyngeal arches. Comparable numbers of BrdU+ cells were observed in wild-type siblings (n=4) and *tbx1* mutants (n=4) at 30 hpf. Scale bar represents 40 μM.

(R and S) Lysotracker Red staining (red) labels dying cells relative to *sox10*:GFP+ neural-crest-derived cells (green) in wild-type siblings (n=6) and *tbx1* mutants (n=6). Arrows show dying cells in the posterior-most pharyngeal arches of a *tbx1* mutant.

(T) Quantification of cell death in *tbx1* mutants. Dying cells were counted in the entire head (from the posterior-most arch to the anterior limit of the embryo, including the brain, arches, and other tissues), as well as in *sox10*:GFP+ cells of the arches. A one-tailed Student's t test with unequal variance was utilized to quantify the number of dying cells, and p values are shown.

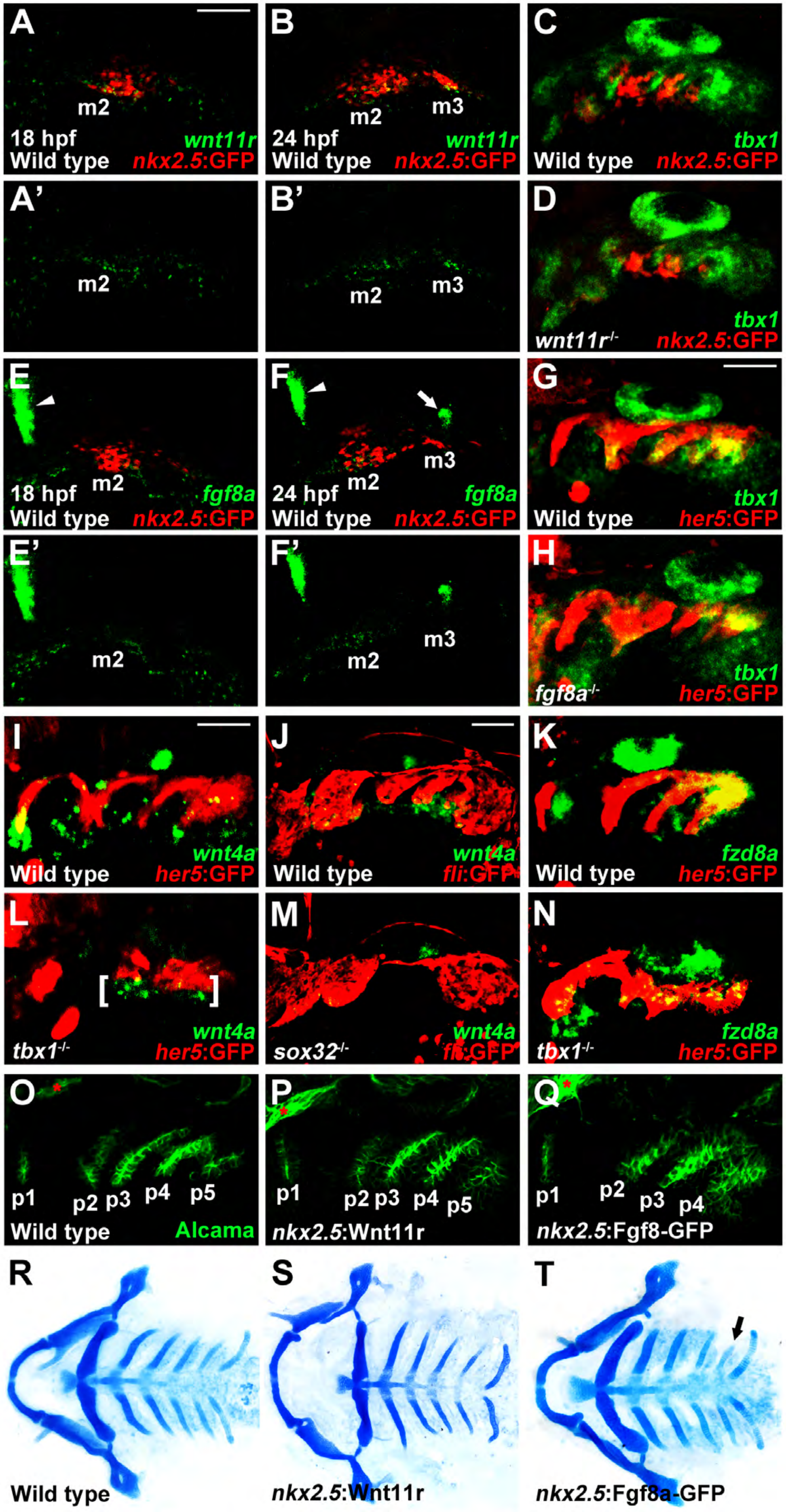
A
m2
18 hpf
Wild type
wnt11r
nkx2.5:GFP
B
m2
m3
24 hpf
Wild type
wnt11r
nkx2.5:GFP
C
Wild type
tbx1
nkx2.5:GFP
A'
m2
B'
m2
m3
D
wnt11r-/-
tbx1
nkx2.5:GFP
E
18 hpf
m2
Wild type
fgf8a
nkx2.5:GFP
F
24 hpf
m2
m3
Wild type
fgf8a
nkx2.5:GFP
G
Wild type
tbx1
her5:GFP
E'
m2
F'
m2
m3
H
fgf8a-/-
tbx1
her5:GFP
I
Wild type
wnt4a
her5:GFP
J
Wild type
wnt4a
fli:GFP
K
Wild type
fzd8a
her5:GFP
L
tbx1-/-
wnt4a
her5:GFP
M
sox32-/-
wnt4a
fli:GFP
N
tbx1-/-
fzd8a
her5:GFP
O
p1 p2 p3 p4 p5
Wild type
Alcama
P
p1 p2 p3 p4 p5
nkx2.5:Wnt11r
Q
p1 p2 p3 p4
nkx2.5:Fgf8-GFP
R
Wild type
S
nkx2.5:Wnt11r
T
nkx2.5:Fgf8a-GFP

Fig. S3, *wnt4a* and *fzd8a* expression in *tbx1* mutants and analysis of *nkx2.5*:Wnt11r and *nkx2.5*:Fgf8a-GFP transgenes

(A-H) Fluorescent in situ hybridization for *wnt11r*, *tbx1,* or *fgf8a* mRNA (green). In red, GFP immunohistochemistry detects *nkx2.5*:GFP+ mesoderm (A-F) or *her5*:GFP+ endoderm (G,H). At 18 hpf, *wnt11r* is expressed in the second arch mesodermal core (m2) (A, n=17), and *fgf8a* is not yet expressed in the *nkx2.5*:GFP+ mesoderm (E, n=21). By 24 hpf, *nkx2.5*:GFP labels the third arch mesodermal core (m3) and *wnt11r* is expressed in both m2 and m3 (B, n=15) and *fgf8a* just in m2 (F, n=20). *fgf8a* mRNA expression is also observed at the midbrain-hindbrain boundary (arrowheads in E and F) and the anterior region of the otic vesicle (arrow in F). At 30 hpf, *tbx1* expression is unaffected in 8/8 *wnt11r* mutants (D) or 11/11 *fgf8a* mutants (H) compared to wild-type siblings (C and G).

(I-N) Fluorescent in situ hybridization for *wnt4a* or *fzd8a* mRNA (green) at 30 hpf. In red, GFP immunohistochemistry detects *her5*:GFP+ endoderm (I,K,L,N) or *fli1a*:GFP+ neural-crest-derived ectomesenchyme (J,M). Compared to wild types (n=61), ectodermal *wnt4a* expression (brackets in L) persists but is disorganized in 18/18 *tbx1* mutants. In contrast, 9/9 *sox32*$^{-/-}$ embryos displayed near complete loss of *wnt4a* ectodermal expression, with the lack of posterior arch segmentation indicative of the absence of pouch endoderm. Compared to normal expression in wild types (n=38), *fzd8a* expression within the *her5*:GFP+ endoderm is largely unaffected in 10/14 *tbx1* mutants.

(O-Q) Alcama immunohistochemistry shows a series of five pouches at 34 hpf. Compared to wild types, we observed normal pouches in 49/49 *nkx2.5*:Wnt11r and 27/36 *nkx2.5*:Fgf8a-GFP embryos. In 9/36 *nkx2.5*:Fgf8a-GFP embryos, we observed delayed and/or disorganized formation of the last pouch. Sensory ganglia are indicated with red asterisks. Anterior is to the left and ventral is down. Scale bar represents 40 μm.

(R-T) Ventral whole-mount views of Alcian-stained facial cartilage. Compared to wild types, 28/28 *nkx2.5*:Wnt11r and 25/31 *nkx2.5*:Fgf8a-GFP embryos formed normal cartilages. In 6/31 *nkx2.5*:Fgf8a-GFP embryos, we observed partial reductions of one ceratobranchial (arrow in T).

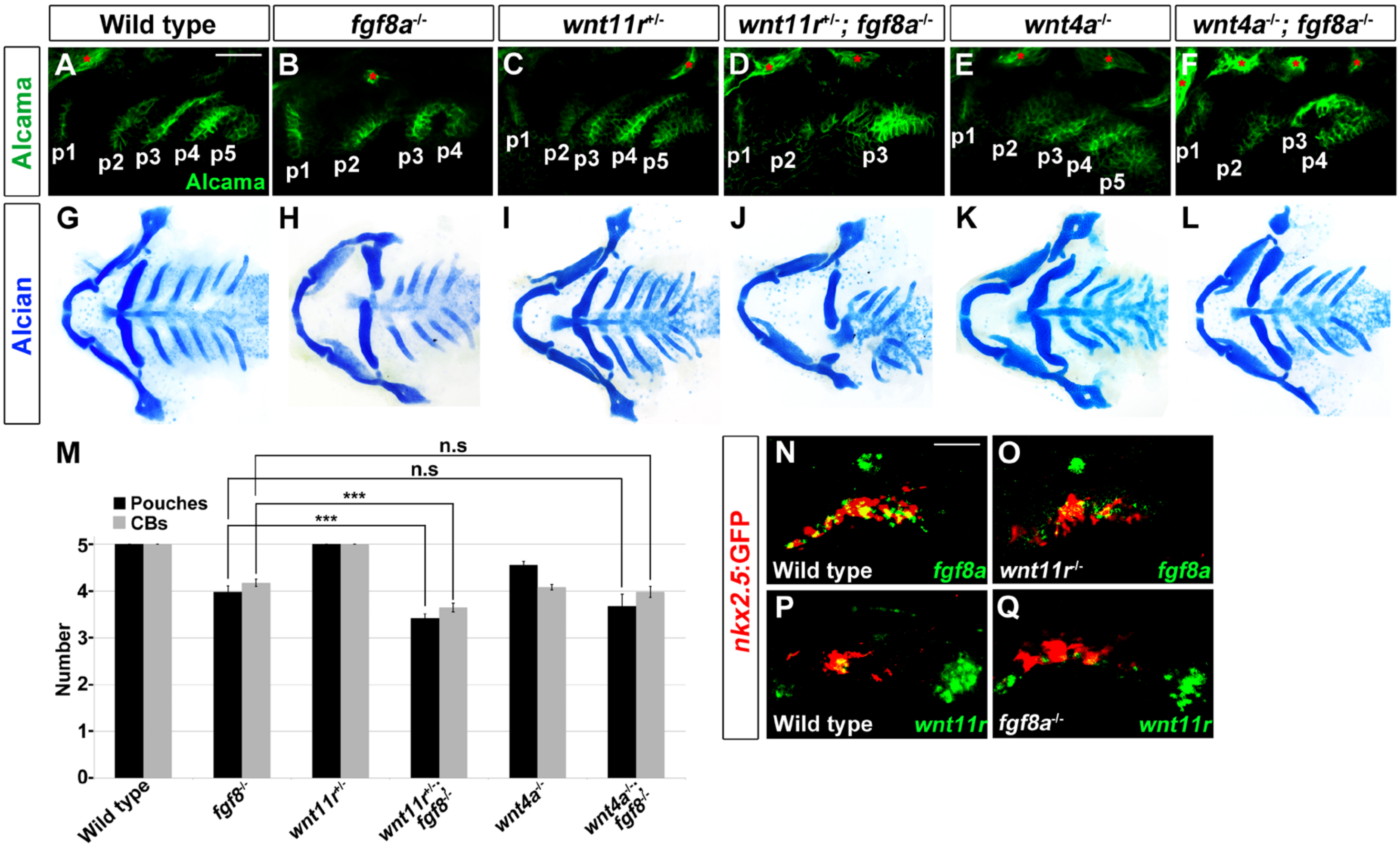

Fig. S4, Interactions between Wnt4a, Wnt11r, and Fgf8a during pouch formation

(A-F) Alcama immunohistochemistry at 34 hpf shows a series of five pouches. Compound *wnt11r*$^{+/-}$; *fgf8a*$^{-/-}$ but not *wnt4a*$^{-/-}$; *fgf8a*$^{-/-}$ mutants show enhancement of pouch defects compared to *fgf8a*$^{-/-}$ embryos alone. Sensory ganglia are indicated with red asterisks.

(G-L) Facial cartilages stained with Alcian. Compound *wnt11r*$^{+/-}$; *fgf8a*$^{-/-}$ mutants but not *wnt4a*$^{-/-}$; *fgf8a*$^{-/-}$ mutants show enhancement of ceratobranchial cartilage defects compared to *fgf8a*$^{-/-}$ mutants alone.

(M) Quantification of pouch and ceratobranchial cartilage (CB) defects. Number of pouches and CBs examined for each genotype: wild type (106, 102), *wnt11r*$^{+/-}$ (51, 47), *wnt4a*$^{-/-}$ (45, 144), *fgf8a*$^{-/-}$ (68, 98), *wnt11r*$^{+/-}$; *fgf8a*$^{-/-}$ (38, 80), and *wnt4a*$^{-/-}$; *fgf8a*$^{-/-}$ (20, 30). Data represent mean ± SEM. ***, $p<0.001$. n.s., not significant. The data for wild type and *fgf8a*$^{-/-}$ are repeated from Figure 4I.

(N-Q) Fluorescent in situ hybridization for *fgf8a* or *wnt11r* mRNA (green) at 30 hpf. GFP immunohistochemistry labels the *nkx2.5*:GFP+ mesoderm in red. *fgf8a* expression is unaffected in 10/13 and slightly reduced in 3/13 *wnt11r* mutants. *wnt4a* expression is unaffected in 15/19 and slightly reduced in 4/19 *fgf8a* mutants. Scale bar represents 40 μM

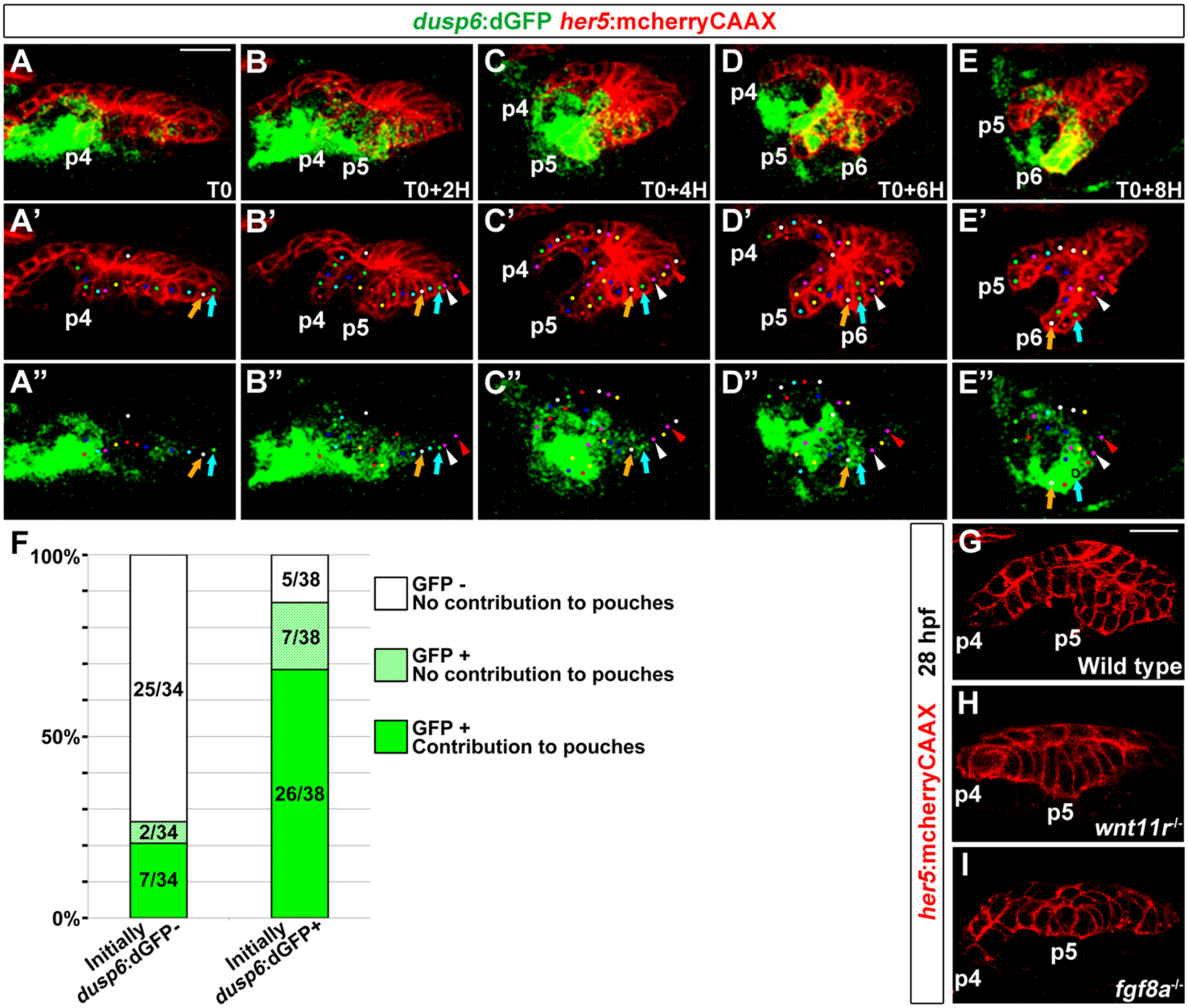

Fig. S5, Preferential contribution of *dusp6*:dGFP+ cells to pouches
(A-E) Representative sections from a time-lapse recording of a wild-type *dusp6*:dGFP; *her5*:mCherryCAAX embryo (see Movie S2) in which individual cells were tracked (colored dots). Orange and blue arrows show two cells that were *dusp6*:dGFP- in the posterior-most domain of pharyngeal endoderm at the start of the recording (T0) and then turned on *dusp6*:dGFP and contributed to the sixth pouch (p6) over the next 8 hours. White and red arrowheads show two adjacent cells that never turned on *dusp6*:dGFP and were excluded from the pouch.

(F) Quantification of the contribution of cells to the pouches based on whether they were initially *dusp6*:dGFP- (left bar) or *dusp6*:dGFP+ (right bar). For initially *dusp6*:dGFP- cells, we then quantified the number of cells that stayed GFP- or turned on *dusp6*:dGFP (GFP+). For initially *dusp6*:dGFP+ cells, we quantified the number of cells that maintained *dusp6*:dGFP (GFP+) or extinguished GFP (GFP-). For both, we also scored the contribution of each category to pouches. Both cells that turned on *dusp6*:dGFP ($p<0.001$) and maintained *dusp6*:dGFP ($p=0.002$) contributed to pouches at a high frequency than cells that remained or turned off *dusp6*:dGFP. y-axis represents % of cells of each category.

(G-I) High magnification confocal sections show *her5*:mCherryCAAX labeling of endodermal cell membranes within the fourth pouch (p4) and presumptive fifth pouch (p5) at 28 hpf. The epithelium of nascent pouch p5 was multilayered in 8/8 wild-type siblings and 5/5 *fgf8a* mutants but only in 1/4 *wnt11r* mutants. Instead, pre-pouch cells in 3/4 *wnt11r* mutants retained a columnar epithelial morphology. Scale bars represent 20 μM.

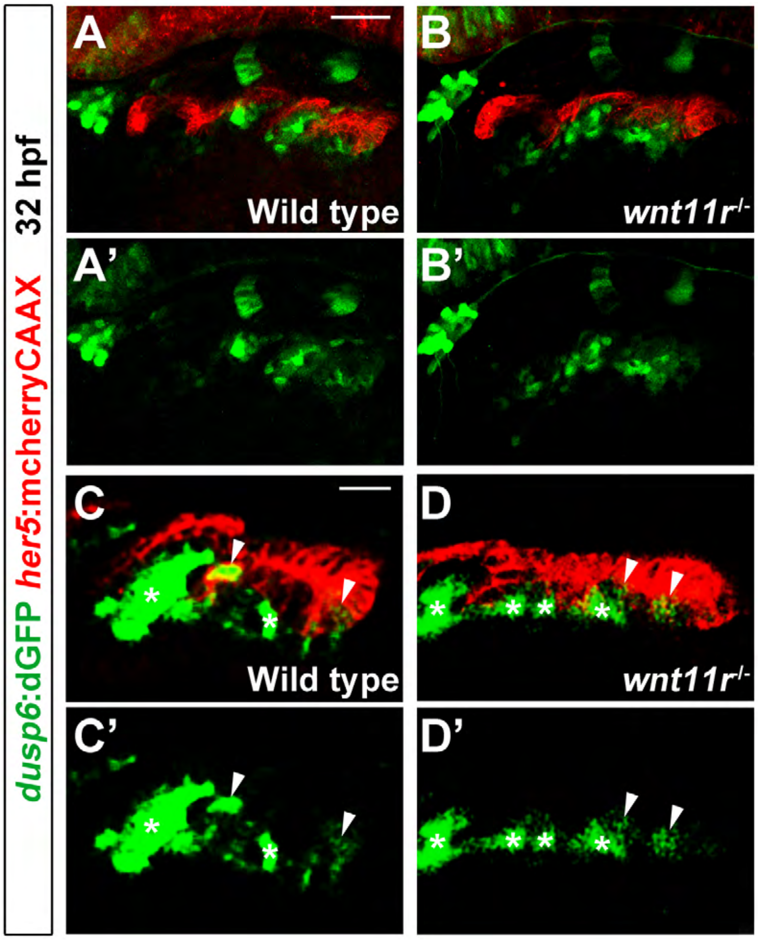

Fig. S6, Fgf activity in the endoderm of *wnt11r* mutants
(A and B) Confocal projections show Fgf activity as marked by *dusp6*:dGFP (green) relative to the *her5*:mCherryCAAX+ pouch endoderm (red) at 32 hpf. In 8/8 *wnt11r* mutants, *dusp6*:GFP expression is grossly unaffected compared to wild-type siblings (n=34). Scale bar represents 40 μM.

(C and D) Higher magnification and gain-enhanced sections focused on the posterior pouches seen in (A) and (B) show *dusp6*:dGFP fluorescence within alternating clusters of endodermal epithelial cells (arrowheads), as well as in clusters of adjacent mesodermal cells (asterisks). While pouch outpocketing is delayed in *wnt11r* mutants, segmental *dusp6*:dGFP fluorescence is still detectable within both the mesoderm and endoderm. Scale bar represents 20 μM.

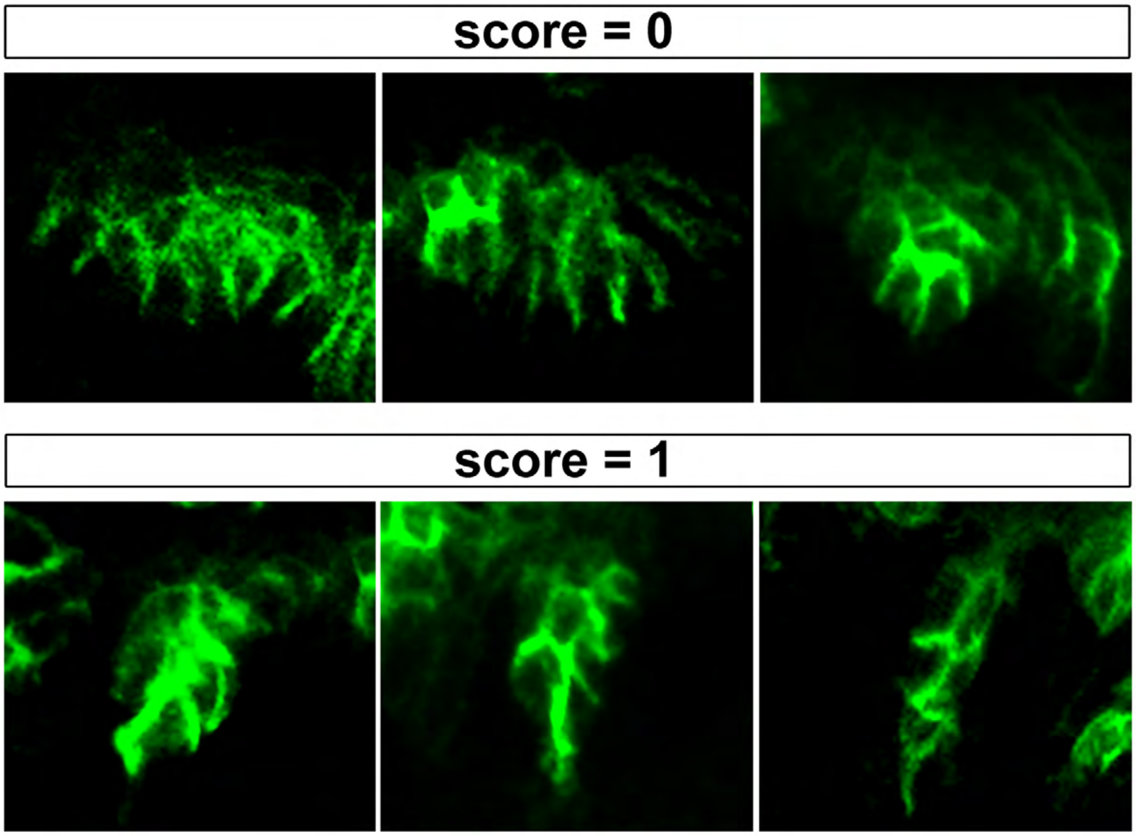

Fig. S7, Criteria for scoring pouch rescue
Several examples of 34 hpf Alcama staining are shown that illustrate lack of pouch rescue (score = 0) or partial/full pouch rescue (score = 1). Pouches were scored as “partial rescue” when they were greater than 50% the length of a corresponding wild-type pouch at that position. These criteria were applied consistently across all experimental groups.

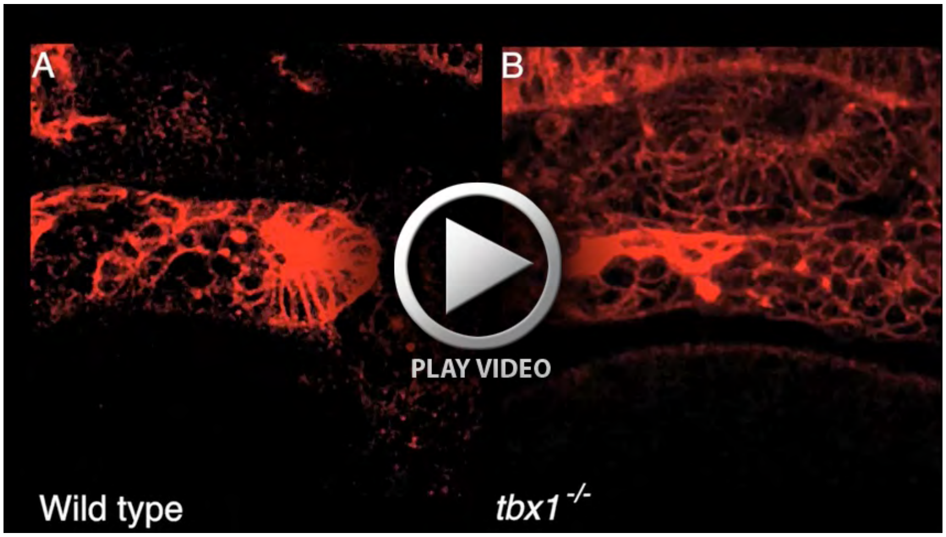

Movie S1. Wild-type and *tbx1*$^{-/-}$ pouch development
In these 24-34 hpf time-lapse recordings, *her5*:mCherryCAAX labels pharyngeal endodermal cell membranes. In the wild-type example (A), sequential formation of the fourth through six pouches is observed. In the *tbx1*$^{-/-}$ embryo (B), *her5*:m-CherryCAAX+ endodermal epithelial cells are present at 24 hpf but fail to display outpocketing behavior during the same period. Lateral is down with anterior to the left.

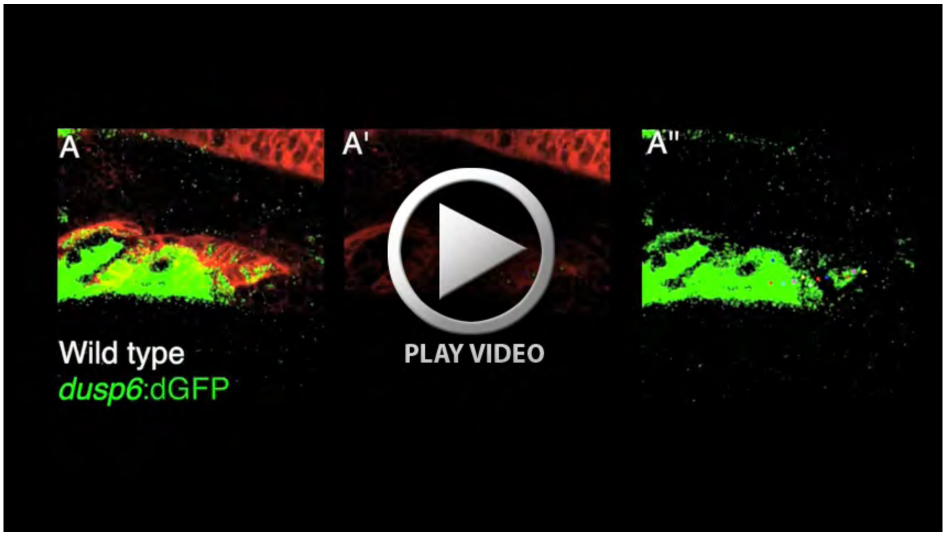

Movie S2. Contribution of *dusp6*:dGFP cells to pouches
In this 24-36 hpf recording of wild-type pouch development, *her5*:mCherryCAAX labels endodermal epithelial cell membranes in red and *dusp6*:dGFP dynamically reports Fgf activity in green. Tracking of endodermal cells (colored dots) allows correlation of *dusp6*:dGFP expression with future contributions to pouches.

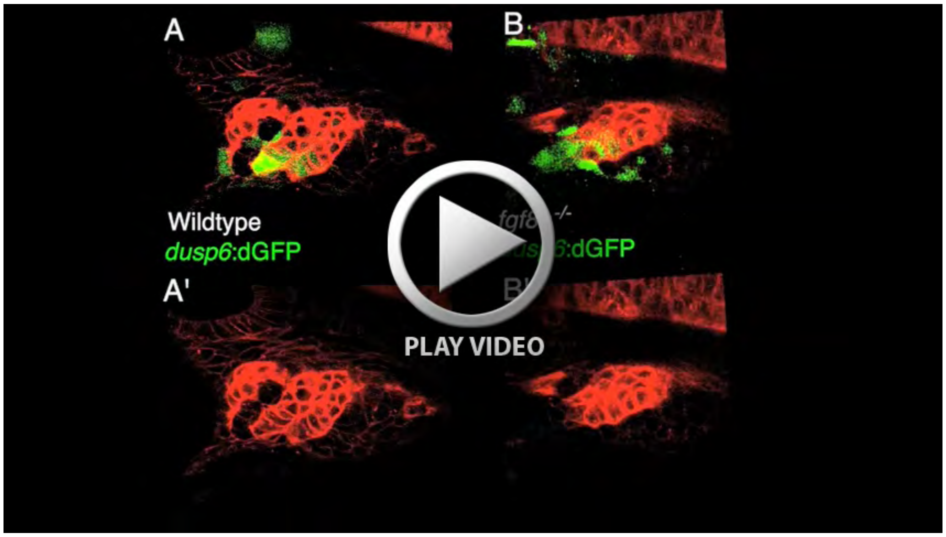

Movie S3. Pouch development in wild types and *fgf8a*$^{-/-}$ mutants
Time-lapse recordings of pouch development from 24 to 34 hpf in embryos expressing *her5*:mCherryCAAX (red) and *dusp6*:dGFP (green). In the wild-type example (B), *dusp6*:GFP fluorescence emergences in largely coherent clusters of cells that contribute to pouches. In contrast, *dusp6*:dGFP fluorescence is patchy in *fgf8a*$^{-/-}$ mutants and pouch formation appears delayed and disorganized. Bottom images show the cell tracking employed for our analyses of endodermal cell velocity and persistence.

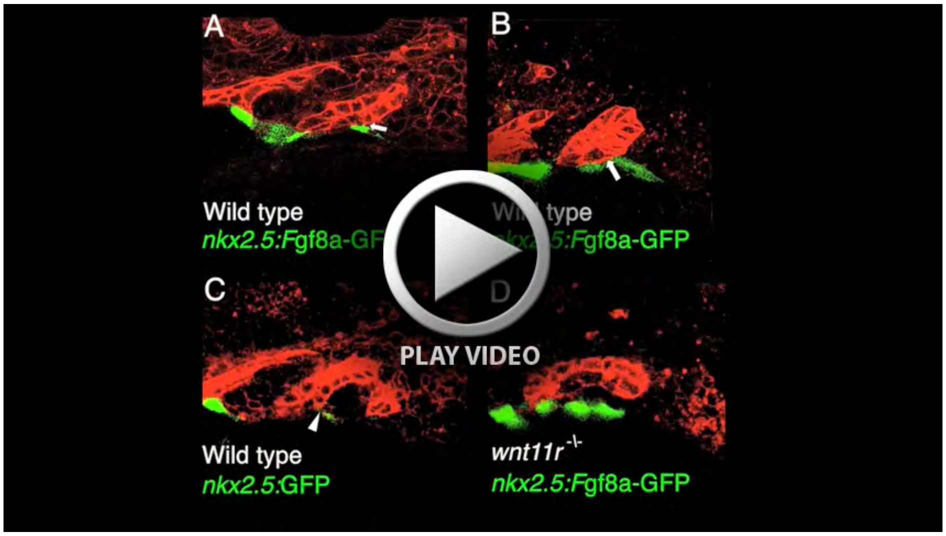

Movie S4. Effect of Fgf8a-GFP misexpression on pouch development in wild types and *wnt11r* mutants
Time-lapse recordings from 25-32 hpf highlight continued development of the third pouch. One-cell-stage embryo injection of transgenes produces mosaic misexpression (green) within the mesoderm. *her5*:mCherryCAAX labels the developing pharyngeal endoderm in red. In A and B, two examples are shown of mosaic misexpression of *nkx2.5*:Fgf8a-GFP in wild types. Arrows highlight the transient diversion of third pouch cells by *nkx2.5*:Fgf8a-GFP+ clones. In C, mesodermal clones expressing a control *nkx2.5*:GFP transgene have no effect on surrounding third pouch cells (arrowheads) in wild types. In D, endodermal cells of *wnt11r*$^{-/-}$ mutants display delayed outpocketing behavior and little response towards ectopic clones of *nkx2.5*:Fgf8a-GFP-expressing mesodermal cells. Single sections are shown for A, C, and E, and thin projections of five sections are shown in B for better appreciation of diverted third pouch cells.
